# Ozone-induced inhibition of kiwifruit ripening is amplified by 1-methylcyclopropene and reversed by exogenous ethylene

**DOI:** 10.1186/s12870-018-1584-y

**Published:** 2018-12-17

**Authors:** Ioannis S. Minas, Georgia Tanou, Afroditi Krokida, Evangelos Karagiannis, Maya Belghazi, Miltiadis Vasilakakis, Kalliope K. Papadopoulou, Athanassios Molassiotis

**Affiliations:** 1 Laboratory of Pomology, Department of Agriculture, Aristotle University of Thessaloniki, University Campus, 54124 Thessaloniki, Greece; 2 Department of Horticulture and Landscape Architecture, Colorado State University, 301 University Avenue, Fort Collins, CO 80523 USA; 3 Institute of Soil and Water Resources, ELGO-DEMETER, 57001 Thessaloniki, Greece; 4 Department of Biochemistry and Biotechnology, University of Thessaly, Biopolis, 41500 Larissa, Greece; 5 UMR 7286 - CRN2M, Centre d’ Analyses Protéomiques de Marseille (CAPM), CNRS, Aix-Marseille Université, Marseille, France

**Keywords:** *Actinidia deliciosa*, Climacteric, Cold storage, Ethylene biosynthesis, Gene expression, Kiwifruit ripening, 1-Methylcyclopropene, Ozone, Postharvest, Proteomics, Softening, Protein-protein interaction

## Abstract

**Background:**

Understanding the mechanisms involved in climacteric fruit ripening is key to improve fruit harvest quality and postharvest performance. Kiwifruit (*Actinidia deliciosa* cv. ‘Hayward’) ripening involves a series of metabolic changes regulated by ethylene. Although 1-methylcyclopropene (1-MCP, inhibitor of ethylene action) or ozone (O_3_) exposure suppresses ethylene-related kiwifruit ripening, how these molecules interact during ripening is unknown.

**Results:**

Harvested ‘Hayward’ kiwifruits were treated with 1-MCP and exposed to ethylene-free cold storage (0 °C, RH 95%) with ambient atmosphere (control) or atmosphere enriched with O_3_ (0.3 μL L^− 1^) for up to 6 months. Their subsequent ripening performance at 20 °C (90% RH) was characterized. Treatment with either 1-MCP or O_3_ inhibited endogenous ethylene biosynthesis and delayed fruit ripening at 20 °C. 1-MCP and O_3_ in combination severely inhibited kiwifruit ripening, significantly extending fruit storage potential. To characterize ethylene sensitivity of kiwifruit following 1-MCP and O_3_ treatments, fruit were exposed to exogenous ethylene (100 μL L^− 1^, 24 h) upon transfer to 20 °C following 4 and 6 months of cold storage. Exogenous ethylene treatment restored ethylene biosynthesis in fruit previously exposed in an O_3_-enriched atmosphere. Comparative proteomics analysis showed separate kiwifruit ripening responses, unraveled common 1-MCP- and O_3_-dependent metabolic pathways and identified specific proteins associated with these different ripening behaviors. Protein components that were differentially expressed following exogenous ethylene exposure after 1-MCP or O_3_ treatment were identified and their protein-protein interaction networks were determined. The expression of several kiwifruit ripening related genes, such as 1-aminocyclopropane-1-carboxylic acid oxidase (*ACO1*), ethylene receptor (*ETR1*)*,* lipoxygenase (*LOX1*), geranylgeranyl diphosphate synthase (*GGP1*), and expansin (*EXP2*), was strongly affected by O_3_, 1-MCP, their combination, and exogenously applied ethylene.

**Conclusions:**

Our findings suggest that the combination of 1-MCP and O_3_ functions as a robust repressive modulator of kiwifruit ripening and provide new insight into the metabolic events underlying ethylene-induced and ethylene-independent ripening outcomes.

**Electronic supplementary material:**

The online version of this article (10.1186/s12870-018-1584-y) contains supplementary material, which is available to authorized users.

## Background

Fleshy fruits undergo a complex developmental program that ends in the irreversible process of ripening and eventual tissue senescence [1]. Over-ripening and ethylene-induced senescence shorten fruit postharvest storage potential and create huge economic losses [2]. Thus, understanding the regulation of fruit ripening is of considerable agronomic value. Kiwifruit (*Actinidia deliciosa*, cv. ‘Hayward’) is classified as climacteric in which ethylene synthesis, perception and signal transduction play key roles in ripening [3, 4]. In climacteric fruits, pre-climacteric exposure to 1-methylcyclopropene (1-MCP), through its high affinity for binding to ethylene receptors can inhibit the perception of ethylene in fruit tissues [5], delay ethylene-dependent ripening and senescence and prolong fruit storage life [6, 7]. Many molecular and genetic mechanisms underlying the action of 1-MCP in fruit ripening have been identified. In kiwifruit, 1-MCP application inhibits expression of specific ethylene receptors (*Ad-ERS1a*, *Ad-ETR2* and *Ad-ETR3*) and several transcription factors (*Ad-ERF4*, *Ad-ERF6*, *Ad-ERF10* and *Ad-ERF14*) [8, 9]. In addition, 1-MCP inhibits kiwifruit softening by reducing cell wall related gene expression, such as polygalacturonase (PG) and expansin (EXP) [10–12]. Together, these data on 1-MCP function indicate that ethylene signal transduction is essential not only to initiate climacteric kiwifruit ripening, but also to complete ripening and senescence.

Ozone (O_3_) can reduce spoilage of fresh fruits and vegetables and delay fruit ripening by directly oxidizing ethylene [13–16]. O_3_ exerts major residual effects in kiwifruit ripening physiology and both ethylene biosynthesis and cell wall turnover are specifically targeted by O_3_ after long-term exposure under cold storage conditions. Two or more months of storage in an O_3_-enriched atmosphere blocked kiwifruit ethylene biosynthesis during ripening at 20 °C by inhibiting ACS and ACO gene expression and enzymatic activity [17, 18]. In agreement with its function as a softening repressor, O_3_ modulated kiwifruit cell wall is remodeled by depressing cell wall swelling, pectin and neutral sugar solubilization and by inhibiting the activity of cell wall-degrading enzymes [18]. Upstream of the ethylene pathway, several transcripts, such as *bet v 1 related allergen*, *3 hydroxy-3-methylglutaryl CoA reductase* and *geranylgeranyl diphosphate synthase* are regulated by O_3_ [19]. The reported proteomic analysis determined that down-regulation of protein expression is one of the components related to kiwifruit ripening inhibition by O_3_, as O_3_ causes widespread down-regulation of protein abundance of ATP-citrate lyase, kiwellin and pectin acetylesterase precursor [17, 19]. Further research is necessary to characterize kiwifruit ripening and the specific roles of O_3_.

This study thoroughly investigates 1-MCP and O_3_ signaling in kiwifruit ripening physiology through a systematic analysis at the physiological and molecular level. Physiological data collected during ripening when fruits were exposed to either ambient air or exogenous ethylene are combined with proteomic and transcriptional approaches.

## Results

### Physiological characterization of the effect of 1-MCP, O_3_ and exogenous ethylene on kiwifruit ripening

**Fig. 1:**
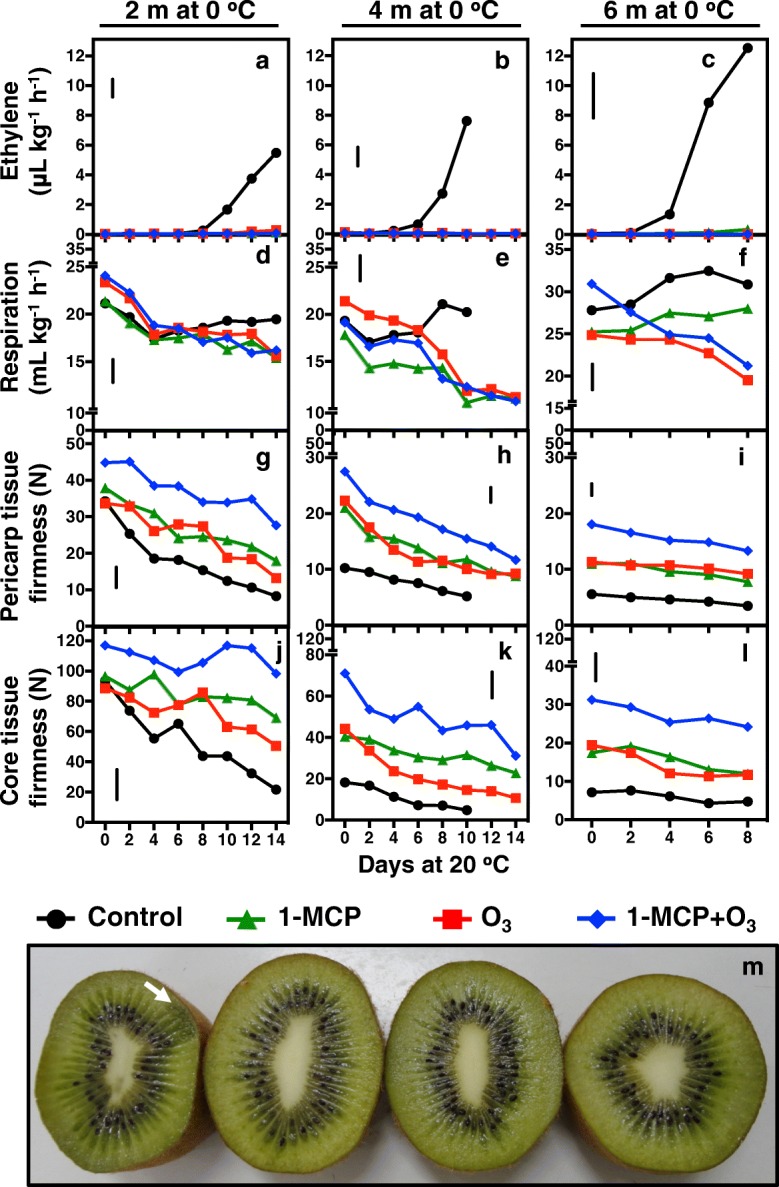
Kiwifruit ripening was inhibited by 1-MCP and O_3_. Following harvest, kiwifruit (cv. ‘Hayward’) were treated with or without (control) 1-MCP (0.6 μL L^− 1^, 0 °C, 24 h) and then cold-stored (0 °C, RH 90%) in two separated cold rooms in which ethylene was controlled by catalytic ethylene oxidation and the atmosphere was ambient (control) or enriched with O_3_ (0.3 μL L^− 1^). Fruits were removed from cold storage after 2, 4 and 6 months and then transferred to 20 °C (90% RH), where kiwifruit ripening was characterized for up to 14 d. Changes in ethylene emission rate (**a**, **e**, **i**), respiration rate (**b**, **f**, **j**), firmness of outer pericarp (**c**, **g**, **k**) and core tissue (**d**, **h**, **l**) in kiwifruit during ripening at 20 °C were measured. Phenotypes of kiwifruit after 8 d ripening at 20 °C following 6 months cold storage (**m**). Values represent the mean of three replicates of 10 fruits each that were analyzed at each ripening time point. Vertical bars in figure plates represent the least significant difference (LSD, *P* = 0.05), which was used for comparisons of means between treatments and ripening time points

**Fig. 2:**
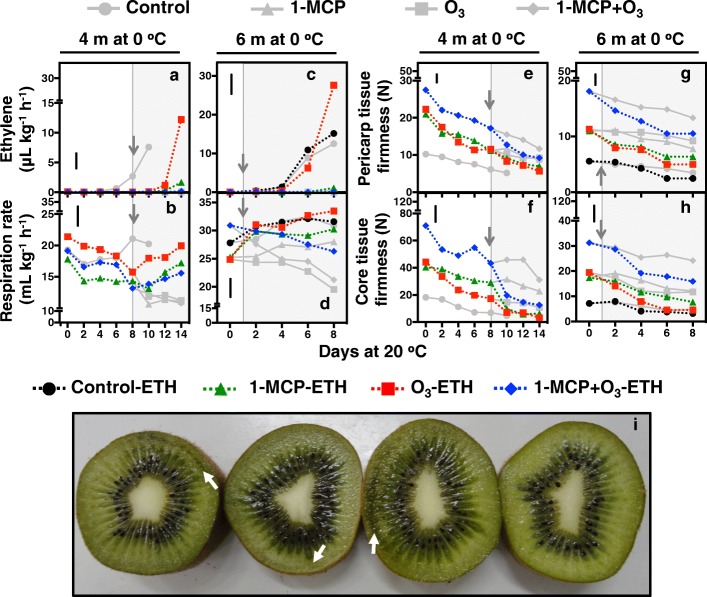
Exposure to exogenous ethylene reversed the O_3_-mediated ripening inhibition in kiwifruit. Following 4 or 6 months of cold storage plus 8 or 1 d maintenance at 20 °C (indicated with grey arrows), respectively, kiwifruit exposed to 1-MCP and/or O_3_ (see Fig. 1) were further treated with exogenous ethylene (100 μL L^− 1^, 20 °C, 90% RH, 24 h) and their ripening was characterized for up to 14 d. Changes during kiwifruit ripening at 20 °C in ethylene emission rate (**a**, **c**), respiration rate (**b**, **d**), firmness of outer pericarp (**e**, **g**) and core tissue (**f**, **h**). Kiwifruit phenotypes at 8 d of ripening at 20 °C following 6 months of cold storage (**i**). Values represent the mean of three replicates of 10 fruits each that were analyzed at each ripening time point. Markers and lines in grey represent kiwifruit untreated with exogenous ethylene as in Fig. 1. Vertical bars in figure plates represent the least significant difference (LSD, *P* = 0.05), which was used for comparisons of means between treatments (exposed or not to exogenous ethylene) and ripening time points

Endogenous ethylene emission from untreated kiwifruit was initiated after 10, 6 and 4 days (d) ripening at 20 °C, following 2, 4 and 6 months of cold storage, respectively. Treatment with 1-MCP immediately after harvest, exposure to O_3_ during cold storage and the combination of both treatments (1-MCP + O_3_) significantly inhibited endogenous ethylene production of kiwifruit during ripening at all three ripening periods (following 2, 4 or 6 months of cold storage) (Fig. 1a-c). To clarify whether the inhibition of endogenous ethylene production observed in kiwifruit exposed to O_3_ and 1-MCP could be recovered by a short treatment with exogenous ethylene, an intermittent experiment was set up. O_3_-stored fruit in the absence of 1-MCP produced significant concentrations of ethylene (12.2 or 27.6 μL kg h^− 1^) after 14 or 8 d ripening in response to exogenous ethylene exposure following 4 or 6 months of cold storage, respectively (Fig. 2a, c). 1-MCP-treated fruit stored without O_3_ exhibited onset of ethylene production (1.7 or 1.1 μL kg h^− 1^) in response to exogenous ethylene after 14 or 8 d ripening at 20 °C following 4 or 6 months of cold storage, respectively (Fig. 2a, c). In contrast, 1-MCP-treated fruit stored in O_3_ atmosphere for up to 4 or 6 months exhibited no detectable ethylene production during ripening following exogenous ethylene treatment (Fig. 2a, c).

Respiration rate (RR) in control fruit immediately upon removal from cold storage following 2, 4 or 6 months was reduced or remained constant after 4 or 2 d ripening, respectively; then RR exhibited a respiratory climacteric increase (Fig. 1d-f). In contrast, the postharvest treatments, with the exception of 1-MCP-treated fruit after 6 months cold storage, reduced RR during ripening following 2, 4 and 6 months of storage. Although RR was unaffected by exogenous ethylene in control fruit during ripening after 6 months of cold storage, it was increased by exogenous ethylene in fruit exposed to O_3_ or 1-MCP individual treatments after 4 or 6 months of cold storage. In contrast, the 1-MCP plus O_3_-treated fruit had the lowest RR after exogenous ethylene treatment (Fig. 2b, d).

Analysis of pericarp and core tissue firmness showed that 1-MCP + O_3_ was the most effective treatment in inhibiting kiwifruit softening during storage (Fig. 1g-l). Interestingly, 1-MCP + O_3_-treated fruit had high pericarp (28 N) and core (98 N) tissue firmness, that is significantly greater than the acceptable for consumption levels (10–15 N), after 2 months of cold storage plus 14 d ripening. Individual 1-MCP and O_3_ treatments lowered the softening rates below those of control fruit; however, they did not retain firmness comparable to 1-MCP + O_3_. 1-MCP alone retained higher levels of core tissue firmness than O_3_-enriched atmosphere (Fig. 1j-l), but there were no differences in pericarp firmness between these two treatments. Exogenous ethylene after 8 d ripening following 4 months of storage increased softening rates in all treatments, although there were sharp changes in core tissue firmness in kiwifruit exposed to both 1-MCP treatments. Following 6 months of cold storage, exogenous ethylene reduced pericarp and core firmness in all treatments while in control fruit, no further firmness reduction was observed (Fig. 2e-h). Kiwifruit from control, control-ETH and O_3_-ETH treatments, which produced high ethylene rates following 6 months of cold storage (Fig. 2a, c), exhibited internal breakdown symptoms due to over-ripening after 8 d (Fig. 2i).

Kiwifruit dry matter content (DMC) remained constant postharvest, irrespective of the experimental conditions (Additional file 1: Figure S1). In addition, soluble solids concentration (SSC) increase was delayed by 1-MCP or O_3_ application while 1-MCP + O_3_ severely delayed SSC accumulation during ripening following 2 months of storage (Additional file 2: Figure S2). Exogenous ethylene, particularly following 4 months of cold storage plus 8 d ripening, increased SSC regardless of treatment (Additional file 3: Figure S3). Generally, no differences were found in titratable acidity (TA) among treatments (Additional file 2: Figure S2), although exogenous ethylene decreased TA in O_3_-treated fruit following 6 months storage plus 8 d ripening (Additional file 3: Figure S3).

### Ethylene biosynthesis during kiwifruit ripening is affected by 1-MCP and O_3_

**Fig. 3:**
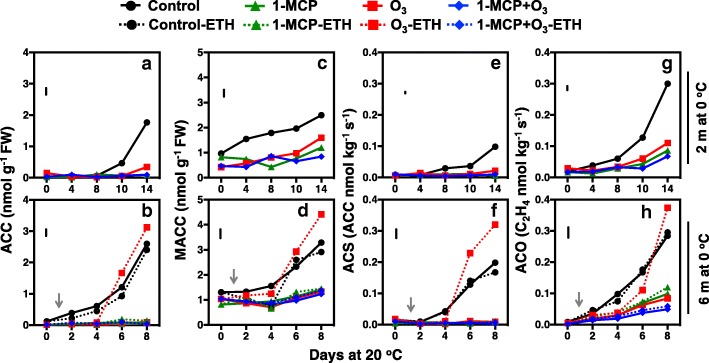
Kiwifruit ethylene biosynthesis was inhibited by 1-MCP and O_3_. Changes in ACC (**a**, **b**) and MACC (**c**, **d**) concentrations and ACS (**e**, **f**) and ACO (**g**, **h**) enzymatic activities in kiwifruit during ripening at 20 °C following 2 or 6 months of cold storage (0 °C, 90% RH). Values represent the mean of three replicates of 10 fruits each that were analyzed at each ripening time point. Vertical bars in figure plates represent the least significant difference (LSD, *P* = 0.05), which was used for comparisons of means between treatments and ripening time points. Arrows indicate exogenous ethylene treatment timing

To further describe the effect of 1-MCP and O_3_ on ethylene biosynthesis, we profiled ethylene biosynthesis intermediates and enzymes (Fig. 3) during ripening at specific time points based on the ripening behavior of control fruit (Fig. 1). These time points included the beginning of ripening immediately upon removal from 2 months of cold storage (0 d at 20 °C), pre-ethylene production (4 d at 20 °C), initiation of ethylene production (8 d at 20 °C), increase of ethylene production (10 d at 20 °C), and peak of ethylene production (14 d at 20 °C). In addition, five similar time points based on the ripening behavior of control fruit (0, 2, 4, 6 and 8 d at 20 °C) were selected following 6 months of cold storage. All postharvest treatments inhibited ACC and MACC concentrations and ACS and ACO enzyme activities during ripening, following 2 and 6 months of cold storage (Fig. 3a-h). Exogenous ethylene after 6 months of cold storage increased ACC concentrations in O_3_-stored fruit at 6 d ripening but did not affect ACC concentrations in control or 1-MCP-treated fruit, compared to their untreated with ethylene counterparts (Fig. 3b). The concentration of ACC in O_3_-treated fruit increased further after 8 d ripening due to exogenous ethylene (Fig. 3b). Ethylene biosynthesis analysis also showed strong inhibition of ACS and ACO activities and ACC accumulation by 1-MCP and O_3_ treatments (Fig. 3a, e, g). Exogenous ethylene following 6 months of cold storage rapidly increased ACS and ACO activities in O_3_-treated (O_3_-ETH) kiwifruit after 8 d ripening (Fig. 3e, g). Exogenous ethylene did not affect ACS and ACO enzyme activities either in control or in 1-MCP-treated fruit with or without O_3_ compared to their untreated with ethylene counterparts (Fig. 3f, h).

### Kiwifruit protein changes and functional classification

**Fig. 4:**
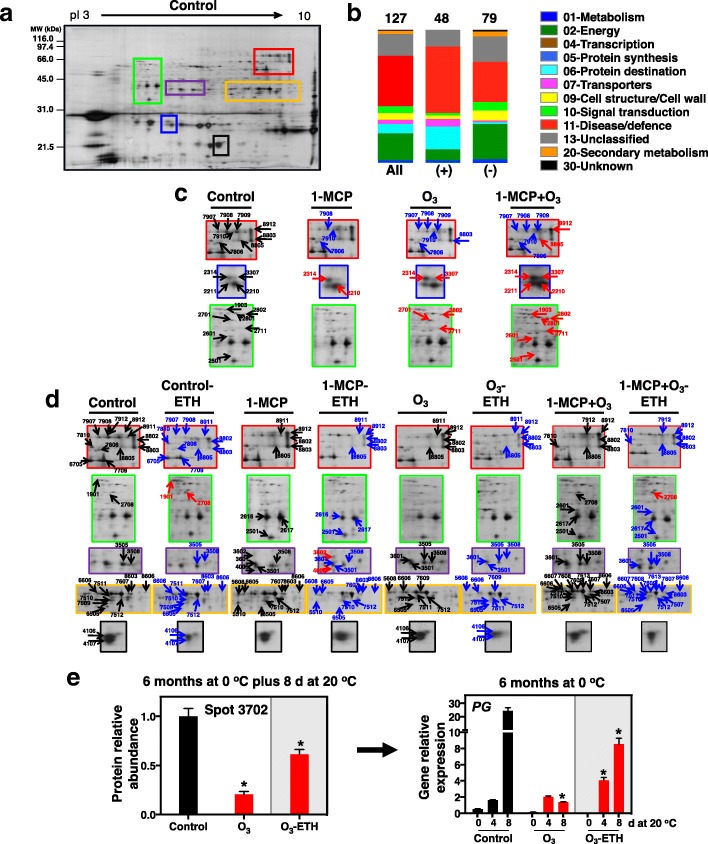
Kiwifruit protein profile gel maps as affected by 1-MCP and O_3_ treatments or exogenous ethylene exposure. Representative 2DE-protein spots of pericarp flesh tissue of control kiwifruit (**a**). Equal amounts (50 μg) of total soluble protein extracts were loaded in each gel. The functional categories of the kiwifruit proteins that identified are shown with color codes (**b**). Enlarged 2DΕ-gel regions exhibiting differences in protein spot abundance as in plate (**a**) for control and 1-MCP-, O_3_- and 1-MCP + O_3_-treated kiwifruit that were cold-stored (0 °C, 90% RH) for 6 months plus 8 d at 20 °C (**c**). Zoom-in areas of the 2DΕ-gel maps exhibiting differences in protein spot abundance of kiwifruit exposed or not to exogenous ethylene (ETH) (**d**). 2DE-maps created three times for a minimum of three independent extractions that each corresponded to a biological replication per treatment. Representative protein spots are listed in Additional file 4: Table S1. Black, red and blue arrows indicate identified proteins that remained unchanged, increased or decreased in abundance, respectively, in control kiwifruit or the ones treated with 1-MCP, O_3_ and 1-MCP + O_3_ and exposed or not to exogenous ethylene. 2DΕ-protein relative abundance of polygalacturonase (PG, Spot 3702, Additional file 11: Table S5) and *PG* expression patterns by real-time RT-qPCR in control and O_3_-treated kiwifruit under external ethylene (ETH) or ambient conditions (**e**). Data (mean ± SE) of three biological replications. Asterisks (*) indicate statistically significant differences between groups (O_3_ vs. control or O_3_-ETH vs. O_3_)

To characterize the current biological system, large scale analysis of kiwifruit proteome was performed in the outer pericarp tissue (green flesh) of kiwifruit stored for 6 months and subsequently ripened 8 d at 20 °C (Figs. 1 and 2). Following comparative 2DE-analysis (Fig. 4a, b) and nanoLC/MS/MS, we identified 127 proteins that differentially accumulated among treatments, of which 48 were up-accumulated and 79 were down-accumulated (Fig. 4b). Identified proteins were predominantly related to disease/defence (39.2%), followed by energy (20.8%), protein destination/storage (7.5%), cell structure/cell wall (5.8%) and signal transduction (5%) (Fig. 4b). A complete list of the protein identification, including peptide sequences, accession number, subcellular localization and matching criteria is presented in Additional file 4: Table S1. Seventeen proteins were identified in more than one spot, indicating that many of the differentially expressed spots were either subjected to post-translational modification or were members of multi-genic protein families. Among these multi-spot identified proteins were β-D-galactosidase (Fig. 4c, spots: 7908, 7909, 7907, 7619, 7910, 8911), chaperonin CPN60 (Fig. 4c, spots: 2801, 2802), fructose-bisphosphate aldolase (Fig. 4d, spots: 6607, 6506, 7510, 6505, 7509, 7608, 6606, 7507), glyceraldehyde 3-phosphate dehydrogenase (GAPDHc, Fig. 4d, spots: 7511, 7513, 7613, 7512, 8606, 3505), lactoylglutathion lyase (Fig. 4c, spots: 2501, 2601), malate dehydrogenase (Fig. 4c, spot: 2314; Fig. 4d, spot: 3501; Fig. 4d, spot: 7609), polyphenoloxidase (Fig. 4d, spots: 3505, 3601), natterin (Fig. 4d, spot: 3508; Fig. 4d, spot: 7709; Fig. 4d, spot: 5510), and remorin (Fig. 4c, spot: 8803; Fig. 4d, spot: 7607).

**Fig. 5:**
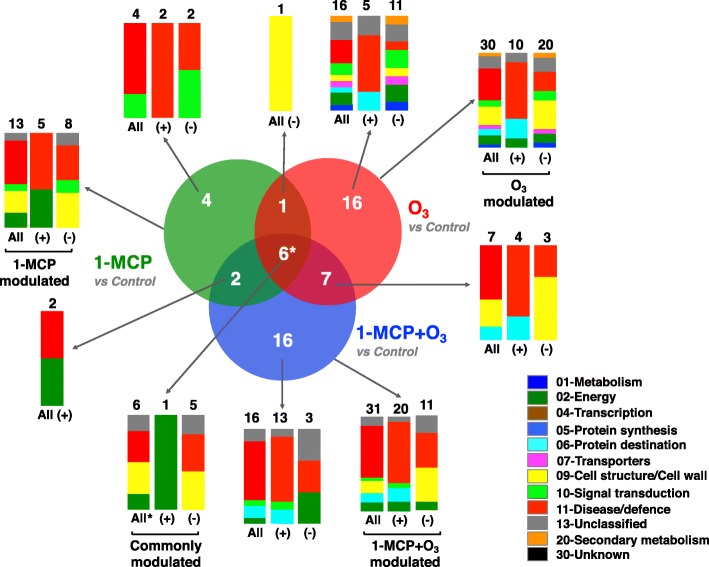
Analysis of differentially accumulated proteins in response to 1-MCP and O_3_ in kiwifruit. Venn diagrams displaying the number of differentially accumulated proteins (54 proteins) in kiwifruit subjected to postharvest treatments (control, 1-MCP, O_3_, or 1-MCP + O_3_). The count of unique or overlapping protein sets is presented. Functional classification and distribution of the identified kiwifruit proteins that changed in abundance due to 1-MCP and/or O_3_ treatments are shown. An asterisk (*) indicates the count of identified kiwifruit proteins commonly regulated by all postharvest treatments. The symbols (+) and (−) indicate identified proteins of kiwifruit that shown up-regulation or down-regulation, respectively

The proteins that exhibited either increased or decreased abundance relative to their respective controls were separated into three groups using a Venn diagram (Fig. 5). The first group is represented by 13 proteins (5 up-accumulated and 8 down-accumulated) that had altered expression with 1-MCP; the second, by 30 proteins (10 up-accumulated and 20 down-accumulated) modulated by O_3_; and the third, by 31 proteins (20 up-accumulated and 11 down-accumulated) regulated by the combination of 1-MCP and O_3_ (Figs. 5 and 7, Additional file 4: Table S1). The functional classes of kiwifruit proteins that were similarly or differentially accumulated in control and 1-MCP/O_3_-treated fruit are also presented (Fig. 5). For example, the 31 proteins modulated by 1-MCP + O_3_ were related to disease/defence, cell structure/cell wall and energy. A proportion of all identified proteins were common to two or more group types. The overlap among all treatments (1-MCP, O_3_, and 1-MCP + O_3_) included 6 proteins, while the overlap between 1-MCP and O_3_ treatments included only 1 protein, polygalacturonase (PG) (Fig. 5, Additional file 4: Table S1). Other proteins were specifically induced or repressed by particular treatments. Four proteins were affected only by 1-MCP, while 16 proteins were exclusively modulated under O_3_ or 1-MCP + O_3_ conditions, suggesting changes in biological activities specific to O_3_ or to the combination of 1-MCP and O_3_. The major portion of 1-MCP/O_3_-specific proteins included disease/defence proteins (Fig. 5).

**Fig. 6:**
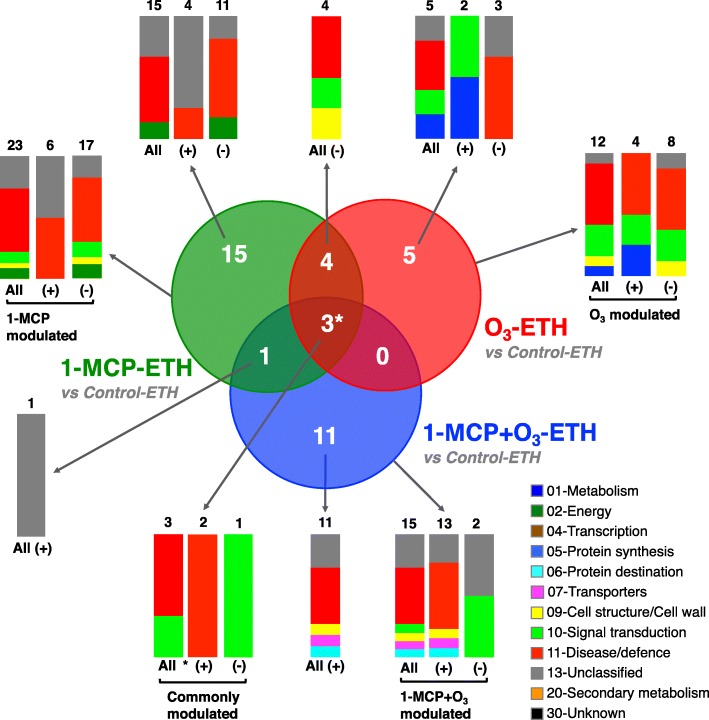
Differentially or commonly accumulated exogenous ethylene-responsive proteins in kiwifruit treated with 1-MCP and O_3_. Venn diagram of 39 proteins differentially expressed between ethylene-exposed treatments (control-ETH, 1-MCP-ETH, O_3_-ETH, 1-MCP + O_3_-ETH) and their counterparts untreated with ethylene (control, 1-MCP, O_3_, 1-MCP + O_3_). The count of unique or overlapping protein sets is presented. Functional classification and distribution of the identified kiwifruit proteins that changed in abundance due to 1-MCP and/or O_3_ treatments are shown. An asterisk (*) indicates the count of identified kiwifruit proteins commonly regulated by exogenous ethylene in all postharvest treatments, compared to their unexposed counterparts. The symbols (+) and (−) indicate identified proteins of kiwifruit that shown up- or down-regulation, respectively

To investigate ethylene-related protein changes, the fruit proteome with each chemical treatment and exogenous ethylene was compared to control fruit treated with ethylene (Fig. 6, Additional file 4: Table S1). Among ethylene-affected proteins, 23 were different in 1-MCP-treated fruit, of which 6 proteins were up-accumulated and 17 were down-accumulated. In addition, 12 differentially accumulated proteins were identified in the O_3_ treatment, 4 up-accumulated and 8 down-accumulated (Figs. 6 and 8, Additional file 4: Table S1). Fifteen proteins (13 up-accumulated and 2 down-accumulated) were modulated by ethylene in the combined 1-MCP + O_3_ treatment (Figs. 6 and 8, Additional file 4: Table S1). Four kiwifruit ETH-responsive proteins were commonly detected in kiwifruit exposed to 1-MCP and O_3_, while 1 protein overlapped between 1-MCP and 1-MCP + O_3_. There was no overlap between O_3_ and 1-MCP + O_3_ treatments. Additionally, 3 proteins were ETH-affected across all treatments. These commonly regulated proteins were involved in disease/defence and signal transduction. A comparison of protein changes in each single treatment (control, 1-MCP, O_3_, or 1-MCP + O_3_) with their ethylene-treated counterparts is also presented (Additional file 5: Figure S4 and Additional file 6: Figure S5).

**Fig. 7:**
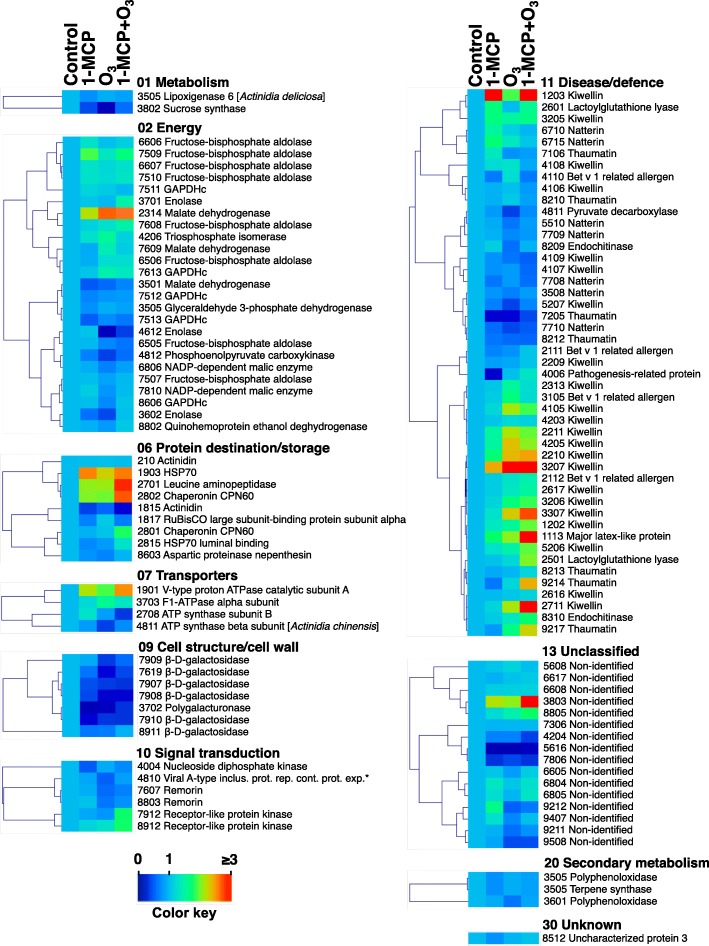
Kiwifruit protein hallmarks were strongly affected by 1-MCP and O_3_. Heat map displaying the relative abundance of each identified protein in treated and control kiwifruits. Fold change means are exhibited using a proportional to the abundance of each identified kiwifruit protein color scale. Means of three biological replications per treatment were presented as ratios between the treatment and control values using the Multi-Experiment Viewer software (version 4.4.1). Relative abundance mean values of the identified proteins are provided in Additional file 2: Table S1. Identified proteins were grouped based on their functional classification as given in Fig. 4. *Viral A-type inclusion protein repeat-containing protein expressed

**Fig. 8:**
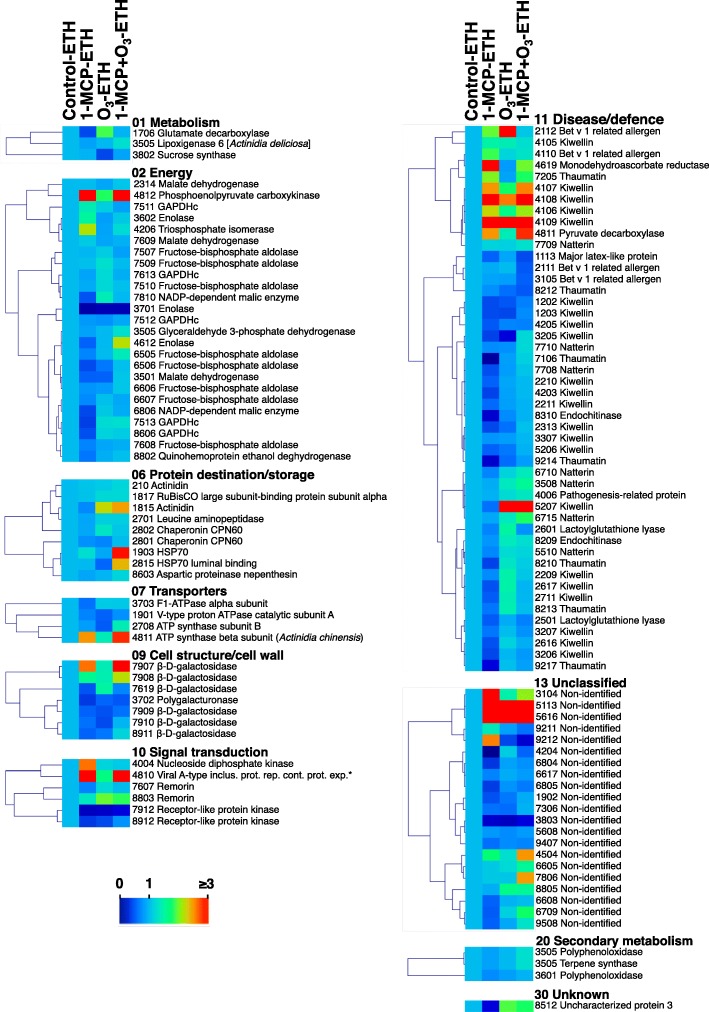
Protein abundance changes in kiwifruit treated with 1-MCP, O_3_ and exogenous ethylene. Heat map displaying the relative abundance of each identified protein compared to the abundance in the untreated with exogenous ethylene control. Fold change means are exhibited using a proportional to the abundance of each identified kiwifruit protein color scale. Means of three biological replications per treatment were presented as ratios between treatment and control values using the Multi-Experiment Viewer software (version 4.4.1). The relative abundance mean values of the identified proteins are provided in Additional file 11: Table S5. Identified proteins were grouped based on their functional classification as given in Fig. 4. *Viral A-type inclusion protein repeat-containing protein expressed

To validate the protein abundance results, transcript expression of *PG* was assayed by q-RT PCR analysis at various ripening times (Fig. 4e). *PG* gene transcript was strongly induced by ripening in control fruit, while O_3_ significantly restricted this *PG* induction, confirmed by the abundance of spot 3702, identified as PG protein. Following exogenous ethylene application, *PG* expression was recovered completely in O_3_-treated fruit (Fig. 4e), supporting the results attained by quantitative 2-DE and LC-MS/MS analysis (Figs. 7 and 8, Additional file 4: Table S1).

### Networks of ripening inhibition- and stimulation-responsive kiwifruit proteins

**Fig. 9:**
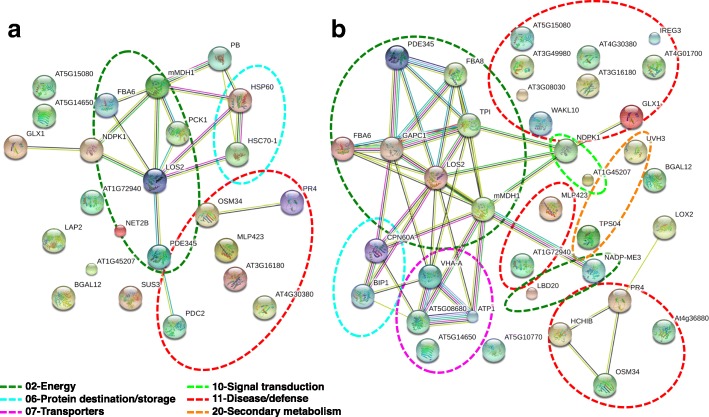
Kiwifruit protein–protein interaction network created by STRING 9.0. Protein-protein interactions are presented for identified kiwifruit proteins undergoing ripening inhibition by 1-MCP and O_3_ (**a**) or ripening induction by exogenous ethylene (**b**). Analysis parameters used were *Arabidopsis thaliana* species and 0.4 confidence level. Lines with different colors represent the different types of evidence used for the predicted associations: gene fusion (red), neighborhood (green), co-occurrence across genomes (blue), co-expression (black), experimental (purple), association in curated databases (light blue), or co-mentioned in PubMed abstracts (yellow). Six groups of protein nodes that are highly interacting are marked with dotted lines and include proteins associated with energy, protein destination/storage, transporters, signal transduction, disease/defence and secondary metabolism functional categories

The experimental structure, with three treatments that inhibited ethylene biosynthesis (1-MCP, O_3_, and 1-MCP + O_3_) and an intermittent test using exogenous ethylene exposure that stimulated ripening, provided an interesting biological system in which to study regulatory networks among the identified proteins. PPI networks created through STRING 9.0 [20] predicted functional links among identified kiwifruit proteins that were expressed in response to ripening inhibitors or stimulators. In kiwifruit treated with ripening inhibitors and not exposed to exogenous ethylene, the major clusters of interacting proteins involve proteins related to energy, protein destination/storage, and disease/defence (Fig. 9a). In kiwifruit treated with ripening inhibitors postharvest and exposed to exogenous ethylene post-storage, the major groups of interacting proteins are related to disease/defence, energy, transporters, protein destination/storage, signal transduction and secondary metabolism (Fig. 9b).

**Fig. 10:**
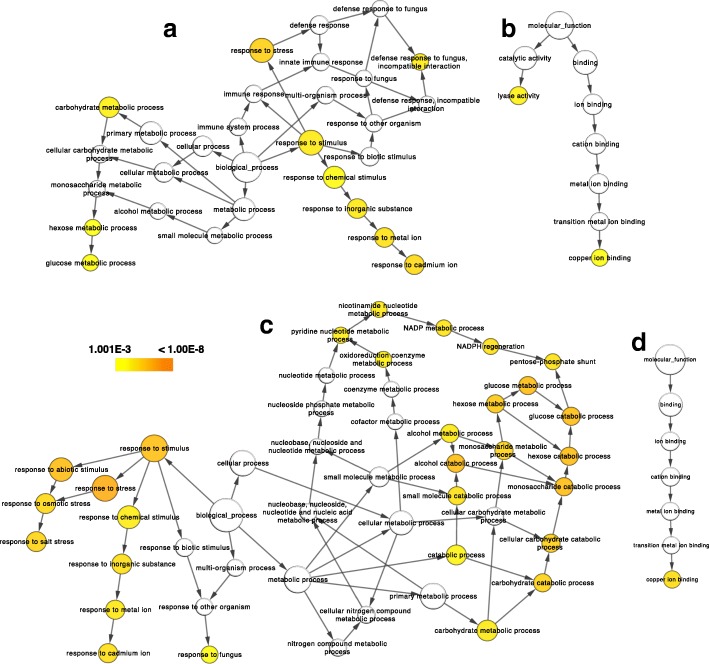
Networks of biological pathways (**a**, **c**) and molecular functions (**b**, **d**) generated by BiNGO. GO categories of kiwifruit TAIR homologous proteins undergoing ripening inhibition by 1-MCP and O_3_ (**a**, **b**) or ripening induction by exogenous ethylene (**c**, **d**). Node size is related to protein counts and color intensity represents the *P*-value for the statistically significant overrepresented GO terms

BiNGO 2.44 [21] software was used to predict statistically significant categories in over- or under-represented biological pathways and molecular functions of kiwifruit undergoing ripening inhibition and stimulation (Fig. 10). The enriched Gene Ontology (GO) lists of biological pathways (Additional file 7: Table S2) and molecular functions (Additional file 8: Table S3) of identified and annotated kiwifruit proteins are provided. The statistically significant over-represented biological pathways in ripening inhibited kiwifruit were the response to stress (*p* = 9.78E-06) and the response to cadmium and metal ion (*p* = 2.53E-05 and 6.34E-05, respectively) (Fig. 10a, Additional file 7: Table S2a). In kiwifruit treated with ethylene in order to stimulate ripening, the major groups were the response to stress (1.44E-09) and glucose catabolism (3.15E-08), along with monosaccharide catabolism (3.41E-08) and hexose catabolism (3.41E-08) (Fig. 10c, Additional file 7: Table S2b). Molecular functions that were enriched in kiwifruit undergoing ripening inhibition were lyase activity (*p* = 3.83E-06) and copper ion binding (*p* = 2.26E-05) (Fig. 10b, Additional file 8: Table S3a). On the other hand, lyase activity (*p* = 3.32E-07) was the most highly enriched molecular function in kiwifruit treated with exogenous ethylene (Fig. 10d, Additional file 8: Table S3b).

### Ripening-related gene and transcription factor expression in kiwifruit exposed to 1-MCP, O_3_ and exogenous ethylene

**Fig. 11:**
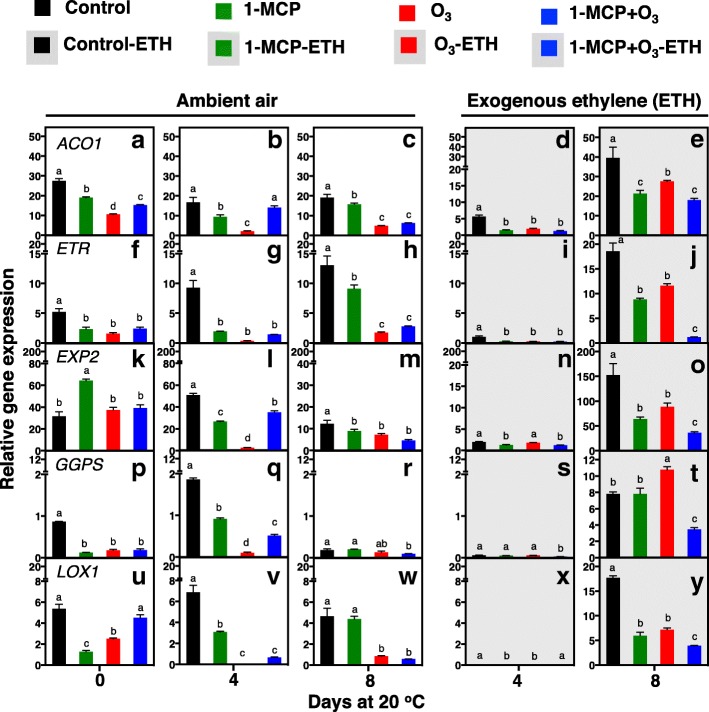
Kiwifruit ripening-related genes and transcription factors expression as affected by 1-MCP, O_3_ and exogenous ethylene. Gene expression of aminocyclopropane-1-carboxylic acid oxidase 1 (*ACO1*, **a**-**e**), ethylene receptor (*ETR*, **f**-**j**), expansin 2 (*EXP2*, **k**-**o**), geranylgeranyl diphosphate synthase (*GGPS*, **p**-**t**) and lipoxygenase 1 (*LOX1*, **u**-**y**) in kiwifruit ripened under ambient air or exogenous ethylene (ETH) at various ripening time points at 20 °C following 6 months of cold storage. Real-time RT-qPCR was used to analyse the relative mRNA abundance on three biological repeats per treatment. Vertical bars in figure plates represent the least significant difference (LSD, *P* = 0.05), which was used for comparisons of means between treatments (exposed or not to exogenous ethylene) and ripening time points

To examine the impact of 1-MCP, O_3_ and exogenous ethylene in kiwifruit ripening, we profiled the expression of several ripening-related genes and transcription factors: aminocyclopropane-1-carboxylic acid oxidase 1 (ACO1), ethylene receptor (ETR), lipoxygenase 1 (LOX1), geranylgeranyl diphosphate synthase (GGPS), and expansin 2 (EXP2) (Fig. 11). Real-time RT*-*PCR analysis showed that *ACO1* expression was constant in the control, but lower in 1-MCP, O_3_ and 1-MCP + O_3_ treatments during ripening following 6 months of storage. Fruit exposed to O_3_ and 1-MCP + O_3_ retained less *ACO1* expression than control or 1-MCP treatments at 8 d ripening (Fig. 11a, b, c). Exogenous ethylene induced *ACO1* transcription in all treatments; however, there was more *ACO1* expression in O_3_-ETH fruit than 1-MCP-ETH and 1-MCP + O_3_-ETH fruit at 8 d (Fig. 11d, e). Additionally, *ETR* expression increased in control fruit at 0 and 4 d and in 1-MCP-treated fruit at 8 d, but not in fruit exposed to O_3_ and 1-MCP + O_3_, that remained suppressed throughout the ripening period (Fig. 11f, g, h). Exogenous ethylene increased *ETR* transcription in control-ETH, 1-MCP-ETH and O_3_-ETH treatments compared to fruit maintained under ambient air (Fig. 11i, j). Meanwhile, *EXP2* expression diminished gradually during ripening, particularly in control and 1-MCP-treated fruits (Fig. 11k, l, m). Exogenous ethylene induced *EXP2* expression in all treatments after 8 d ripening compared to fruit maintained under ambient air. Control-ETH followed by O_3_-ETH and 1-MCP-ETH conditions exhibited the most *EXP2* expression after 8 d ripening, with 1-MCP + O_3_-ETH significantly lower (Fig. 11n, o). In all treatments except O_3_, *GGPS* expression was induced at 4 d; however, *GGPS* declined to basal levels at 8 d (Fig. 11p, q, r). Exogenous ethylene provoked *GGPS* expression in all treatments at 8 d. Fruit exposed to O_3_-ETH exhibited the most *GGPS* expression after 8 d ripening, followed by control-ETH and 1-MCP-ETH, with 1-MCP + O_3_-ETH significantly less (Fig. 11s, t). Gene expression of *LOX1* in fruit treated with O_3_ or 1-MCP + O_3_ after 4 and 8 d ripening was less than control and 1-MCP (Fig. 11u, v, w). Exogenous ethylene stimulated *LOX1* expression in all treatments after 8 d ripening. Control-ETH had the most *LOX1* expression at 8 d, followed by O_3_-ETH and 1-MCP-ETH, with 1-MCP + O_3_-ETH having the least (Fig. 11x, y).

## Discussion

This study was designed to describe and evaluate physiological and molecular changes caused by O_3_ and 1-MCP in kiwifruit. The underlying rationale was to provide data relevant to a comprehensive understanding of regulatory mechanisms governing climacteric fruit ripening.

### Combined 1-MCP and O_3_ treatment severely inhibits kiwifruit ripening

Our results on kiwifruit ripening after 1-MCP and O_3_ treatment (Fig. 1a-f) are consistent with previous work indicating that postharvest 1-MCP treatment and cold storage in an atmosphere enriched with O_3_ can effectively inhibit ethylene emission rate and RR during kiwifruit ripening [10, 12, 17–19]. Although fruit exposed to 1-MCP or O_3_ alone showed similar ripening behavior based on ethylene emission (Fig. 1a-c) and pericarp firmness (Fig. 1g-i), there were substantial differences in RR (Fig. 1d-f) and core tissue firmness (Fig. 1j-l). These differences indicate that 1-MCP or O_3_ could regulate kiwifruit ripening through ethylene-dependent and independent pathways. This hypothesis was further supported by the combined 1-MCP + O_3_ treatment, which suppressed kiwifruit softening more than the individual treatments alone (Fig. 1g-l). From a practical perspective, treatment with 1-MCP and subsequent cold storage in an O_3_-enriched atmosphere could effectively inhibit kiwifruit ripening and softening and successfully extend their storage potential by 1.5 to 2 more months beyond individual 1-MCP or O_3_ application. This is the first report of a positive interaction between 1-MCP and O_3_ in fruit postharvest physiology and represents an interesting experimental model for fruit ripening syndromes. The severe ripening inhibition by 1-MCP + O_3_ could lead to failure of the fruits ability to ripen and soften at eating-ripe firmness, particularly following short-term cold storage (2 months or less). It is recommended, therefore, that this combined 1-MCP + O_3_ treatment should be applied only on fruit intended for long-term cold storage (more than 3 months) to avoid possible consumer rejection. Alternatively, 1-MCP + O_3_-treated fruits should be exposed to exogenous ethylene if they have to be transported to the market earlier than the three-month storage window.

### Ripening inhibition is reversible by exogenous ethylene in O_3_-treated, but not in 1-MCP + O_3_-treated, kiwifruit

The mechanism of 1-MCP action is well-characterized and involves tight binding to ethylene receptors in plant tissues, blocking ethylene signaling [5]. Although O_3_ inhibits ripening [17], the mechanism has not been clearly defined. The critical question raised by this and previous studies [17, 18] is whether the mode of action of O_3_ in kiwifruit ripening stems from permanent oxidative damage or from potentially reversible biochemical inhibition. To directly address this question and given that exogenous ethylene directly induces kiwifruit ripening [4, 22], 1-MCP/O_3_–treated kiwifruit that could not produce ethylene were exposed to exogenous ethylene (ETH) at specific ripening times. ETH treatment did not induce rapid endogenous ethylene production in fruit treated with 1-MCP, unlike in control-ETH-treated fruit (Fig. 2a, c). This suggests that 1-MCP blocks normal feedback regulation of ethylene production in kiwifruit, as in mature banana fruit [23]. On the other hand of particular interest is that O_3_-treated fruit exposed to exogenous ethylene produced more endogenous ethylene (Fig. 2a, c), and can therefore sense and transduce ethylene signalling. The significant ethylene emission levels from O_3_-ETH treated fruit suggests that O_3_ may block kiwifruit ripening through biochemical inhibition of ACS and ACO activity, rather than through oxidative damage to some ethylene biosynthesis pathway components (Fig. 3).

### Specifically accumulated proteins by postharvest treatments provide insights into understanding kiwifruit ripening

To further explore fruit ripening inhibition by chemical treatments (Figs. 1, 2 and 3), we used 2DE coupled with MS/MS to characterize 1-MCP and/or O_3_ proteome activity. Thirteen or 30 proteins changed in response to 1-MCP or O_3_, respectively, while 31 proteins were altered when these treatments were combined (Fig. 5, Additional file 4: Table S1). Nucleoside diphosphate kinase, a possible component of the ethylene signal transduction chain [24], was down-accumulated by 1-MCP only (Fig. 7). A similar down-regulation of this protein has been reported in response to postharvest 1-MCP treatment in papaya fruit [25]. The down-accumulation of a pathogenesis-related (PR) protein exclusively by 1-MCP suggests that PR may be associated with ethylene-related susceptibility to pathogen infection. A similar trend in PR abundance was observed in unripe banana fruit exposed to 1-MCP [26]. The observed up-regulation of two kiwellin isoforms by 1-MCP, combined with several other kiwellin isoforms that were up-accumulated by the various 1-MCP/O_3_ treatments and the reversal of this up-regulation by exogenous ethylene, (Fig. 8, Additional file 4: Table S1) suggests that kiwellin has an important role in kiwifruit ripening.

O_3_ probably acted as a protein repressor, since most proteins exclusively affected by O_3_ were down-accumulated (Fig. 7). Kiwifruit metabolism is unusual in that carbon is primarily stored as starch and eventually, through the ripening process, is hydrolyzed and converted to CO_2_ and sugars [27]. To accurately estimate a carbon budget in kiwifruit during ripening, it is necessary to consider CO_2_ production through respiration and sugar accumulation, which represent the glycolytic and gluconeogenic carbon flux, respectively. Both respiration and the expression of protein-associated gluconeogenesis-glycolysis, such as phosphoenolpyruvate carboxykinase, sucrose synthase and enolase, were depressed by O_3_ (Fig. 7). This finding suggests that carbon can be diverted in gluconeogenic (sucrose biosynthesis) and at the same time in glycolytic (CO_2_ production) pathways in kiwifruit undergoing ripening inhibition. This is consistent with the patterns of malate dehydrogenase abundance described below and collectively suggests that additional regulators besides endogenous ethylene may govern kiwifruit ripening inhibition. In kiwifruit, remorins and ‘Viral A-type inclusion protein repeat-containing protein expressed’ were induced by ripening and reduced by O_3_ [4, 19]. Because the above-mentioned proteins were specifically depressed by O_3_ (Fig. 7), down-accumulation of these proteins plays a role in kiwifruit ripening inhibition.

Our proteomic analysis revealed accumulation of several kiwifruit proteins (*n* = 16) in response to combined 1-MCP and Ο_3_ (Figs. 5 and 7, Additional file 4: Table S1). Accumulation of several intracellular chaperones, such as HSP 70 and chaperonin CPN60, in fruit exposed to 1-MCP + Ο_3_ (Fig. 7) may be associated with its improved postharvest performance after long cold storage, since chaperones promote fruit cell survival following cold stress [28]. A receptor-like protein kinase (RLK) and a major latex-like protein (MLP) were specifically up-accumulated by 1-MCP + Ο_3_ and this response was reversed by exogenous ethylene (Fig. 8, Additional file 4: Table S1). Plant RLKs are transmembrane proteins that perceive signals of environmental conditions and developmental status through their extracellular domains and propagate the signals via their intracellular kinase domains [29]. MLP proteins belong to the Bet v 1 family which act through binding ligands such as cytokinins, brassinolides or secondary metabolites, and trigger downstream signal transduction [30]. The observed changes in abundance of specific isoforms of Bet v 1-related allergen caused by either O_3_ or 1-MCP + O_3_ (Fig. 7, Additional file 4: Table S1) provide further insights for a potential role of Bet v 1-related allergen during kiwifruit ripening, as was previously reported [4]. 1-MCP + O_3_ induced two isoforms of lactoylglutathione lyase (Fig. 7, Additional file 4: Table S1) that were depressed by subsequent exposure to exogenous ethylene (Fig. 8, Additional file 4: Table S1). Lactoylglutathione lyase participates in glutathione-based detoxification of methylglyoxal (methylglyoxal pathway) and was identified via proteomic analysis in various plant systems [31]. This finding and the fact that glyceraldehyde 3-phosphate dehydrogenase, also involved in the methylglyoxal pathway, was affected by exogenous ethylene (Fig. 8, Additional file 4: Table S1) are consistent with reports that involve this pathway with fruit ripening [32, 33], as a potential mechanism to control methylglyoxal levels [34].

Although the two single treatments (1-MCP and O_3_) induced similar inhibition of ethylene biosynthesis, there was little overlap in protein signatures among them (Fig. 5), demonstrating that these substances may inhibit kiwifruit ripening through different pathways. However, there is also evidence that they use similar mechanisms to inhibit ripening. Accumulation of polygalacturonase (PG) and beta-D-galactosidase decreased in kiwifruit exposed to either 1-MCP or O_3_ (Fig. 7), which is consistent with greater firmness retention (Fig. 1g-l**)** and the *PG* expression pattern (Fig. 4e), thus indicating that repression of cell wall remodeling by these chemicals is a critical control point to prevent kiwifruit softening. An isoform of malate dehydrogenase and natterin were regulated by all postharvest treatments (Fig. 7). Malate dehydrogenase is involved in gluconeogenesis in fruit [35] and our results indicate that ripening inhibition may promote gluconeogenesis by inducing malate dehydrogenase. Natterin-like proteins are pore-forming, highly toxic complexes, that may be associated with the defence mechanism of specific animals [36]. Although the active role of natterin in higher plants is unknown, our previous study found this protein up-regulated in kiwifruit undergoing artificial ripening [4]. However, the mechanisms underlying the function of natterin during kiwifruit ripening remains unclear.

### Regulatory networks of 1-MCP/O_3_- and ethylene-responsive proteins of kiwifruit

Although proteins might be isolated from living cells, usually they do not function as single entities, but rather form complexes which are essential to various cellular processes [37]. Bioinformatic analysis using STRING 9.0 [20] highlighted kiwifruit protein-protein interaction networks of the identified proteins expressed differential regulation patterns by ripening inhibition or induction (Fig. 9). In kiwifruit experiencing ripening inhibition, the major group contained proteins that are associated with energy, such as enolases, which can interact with the group of disease/defence-associated proteins (e.g., pyruvate decarboxylase) and the group of protein destination/storage-associated proteins (e.g., HSP70) (Fig. 9a, Additional file 9: Table S4). Enolase (LOS2 in Fig. 9, Additional file 9: Table S4), was defined as the central core protein in the created interacting network among the identified proteins of the present study. Enolase, an enzyme which is also called phosphopyruvate hydratase, is responsible for the catalysis of 2-phosphoglycerate (2-PG) conversion to phosphoenolpyruvate (PEP), in the ninth step of glycolysis. The down-accumulation of two enolase isoforms by the two kiwifruit ripening inhibitors O_3_ and 1-MCP and their induction by exogenous ethylene (Figs. 7 and 8; Additional file 4: Table S1) reveals a potential association of enolase in kiwifruit climacteric ripening regulation. The above observation of increased enolase accumulation during fruit ripening is in agreement with previous reports of kiwifruit and tomato fruit experiencing ripening as a consequence of exogenous ethylene exposure [4, 38]. In kiwifruit exposed to exogenous ethylene, the major group of energy-associated proteins interacts with five other protein clusters: disease/defence-associated (e.g., lactoylglutathione lyase), protein destination/storage-associated (e.g., HSP70 luminal binding), transporters-associated (e.g., F1-ATPase alpha subunit), signal transduction-related (nucleoside diphosphate kinase), and secondary metabolism-associated (terpene synthase), further highlighting the importance of ethylene in kiwifruit ripening (Fig. 9b, Additional file 9: Table S4).

Bioinformatic analysis using BiNGO [21] was able to predict the major molecular functions of the identified proteins that are altered in kiwifruit experiencing ripening inhibition or induction, including lyase activity (5 proteins) and copper ion binding (4 proteins) in the former but only copper ion binding (6 proteins) in the latter (Fig. 10b, d, Additional file 8: Table S3). Bioinformatic analysis also indicated that stress response was the most affected biological pathway in kiwifruit treated with ripening inhibitors (Fig. 10c, Additional file 7: Table S2). Thirteen proteins were classified in the general category of response to stress, including phosphoenolpyruvate carboxykinase, pyruvate decarboxylase, malate dehydrogenase, HSP 70, fructose-bisphosphate aldolase, nucleoside diphosphate kinase, enolase, pathogenesis-related protein, and others (Fig. 10c, Additional file 7: Table S2a). Three other biological pathways that were over-expressed in kiwifruit experiencing ripening inhibition were the response to cadmium ion (6 proteins), response to metal ion (6 proteins), and response to stimulus (13 proteins). In kiwifruit experiencing ripening as a result of exogenous ethylene, the biological pathways over-represented included stress response (16 proteins), glucose catabolism (5 proteins), monosaccharide catabolism (5 proteins), and hexose catabolism (5 proteins) (Fig. 10d, Additional file 7: Table S2b).

### Both 1-MCP and O_3_ regulate expression of ripening-related gene and transcription factors

In addition to the protein changes described above, a crucial set of genes actively involved in kiwifruit ripening [3, 9, 10] was investigated. Notably, 1-MCP and O_3_ treated fruits showed differences in expression of several ripening-related genes (Fig. 11), suggesting that these endogenous ethylene inhibitors act largely independently. The *GGPS* reaction produces geranylgeranyl diphosphate (GGPP), a common precursor for the synthesis of phyllochinones, tocopherols, plastoquinones, chlorophylls, carotenoids, gibberellins, and other hormones [39]. *GGPS* (Fig. 10p-r, s, t), *ACO1* (Fig. 10a-c, d, e) and *ETR* (Fig. 11f-h, i, j) expression was strongly depressed by O_3_ and stimulated by exogenous ethylene. This suggests that exogenous ethylene not only acts downstream of ethylene synthesis and signaling, but also as a regulator of various isoprene-containing ripening compounds in O_3_-treated fruits. *EXP2*, which is involved in cell wall-loosening [40], exhibited a pattern of induced expression similar to *PG* in O_3_-treated fruit exposed to exogenous ethylene (Fig. 4e). Ethylene-induced *EXP2* expression might affect access of hydrolases to cell wall polymers and promote cell wall disassembly and the subsequent fruit softening [41], thereby acting with PG to fine-tune cell wall metabolism in ripening O_3_-treated kiwifruit. In contrast, expression of *ETR*, *EXP2*, *GGP* and *LOX1* under exogenous ethylene remained lower in fruit exposed to 1-MCP + O_3_ (Fig. 10), consistent with the displayed ripening inhibition (Figs. 1 and 2), most likely as a consequence of decreased ethylene sensitivity under such conditions (Fig. 3). The differences in 1-MCP- and O_3_-dependent ripening inhibition are further supported by the contrasting *LOX1* pattern during kiwifruit ripening in ambient air (Fig. 10). *LOX1*, which catalyzes hydroperoxidation of polyunsaturated fatty acids, regulates fruit ripening through ethylene*-*dependent and -independent pathways [42, 43]. These data suggest that *ACO*, *ETR*, *LOX1*, *GGP*, *PG* and *EXP2* are associated with kiwifruit ripening regulation and provide new perspective on understanding 1-MCP- and O_3_-mediated ripening inhibition.

## Conclusions

This is the first study that shows the combination of 1-MCP and O_3_ severely inhibits ethylene production and reduces softening rates in kiwifruit, leading to depressed ripening even under exogenous ethylene exposure. Endogenous ethylene biosynthesis inhibition in kiwifruit by long-term exposure to O_3_-enriched cold storage is reversible by post-storage exogenous ethylene exposure. Protein and gene expression analysis showed that 1-MCP and O_3_ have both common and (mostly) unique roles in kiwifruit ripening. Taken together, our results provide a physiological basis for future research on the implications of both 1-MCP and O_3_ in climacteric fruit ripening.

## Methods

### Fruit material, postharvest treatments and experimental approach

‘Hayward’ kiwifruit grown in a commercial orchard (Naousa, Central Macedonia, Greece) were harvested at the stage of physiological maturity (fresh weight: 93.4 ± 2 g; pericarp tissue firmness: 63.8 ± 1.9 N; core tissue firmness: 144.5 ± 5.4 N; SSC: 7.6 ± 0.2%; TA (citric acid, %): 1.9 ± 0.1%; and DMC: 17.4 ± 0.5%). Fruits were divided randomly into lots (113 lots) of 30 fruits. The initial quality of kiwifruit was analyzed in one lot immediately after harvest and the rest were split into two groups (56 + 56) and treated with or without 1-MCP (0.6 μL L^− 1^ SmartFresh, AgroFresh Inc., Rohm and Haas, Spring House, PA, USA) for 24 h at 0 °C using a 4000-L airtight tent in the cold room, according to manufacturer’s instructions. Immediately after 1-MCP treatment, fruits of both groups were cold-stored (0 °C, 95% RH) in two separate cold rooms in which ethylene was oxidized by catalytic ethylene oxidation (Swintherm model BS 500, Fruit Control Equipments s.r.l., Milano, Italy). Cold room atmosphere was ambient (control) or enriched with 0.3 μL L^− 1^ ozone through a dedicated system of continuous ozone generation and monitoring (oxygen generator model SEP-100, ozone generator model COM-AD-04 and ozone analyser model MP-6060, Anseros Klaus Nonnenmacher GmbH, Tübingen, Germany). Ethylene concentration in the cold storage rooms was monitored and was below the accepted threshold concentration for commercial kiwifruit storage (10 nL L^− 1^) and not significantly different from each other (data not shown). After 2, 4 and 6 months, fruits were removed from cold storage and held at 20 °C (90% RH), where kiwifruit ripening was analyzed either immediately, or after 2, 4, 6, 8, 10, 12 and 14 d.

Postharvest treatments were segregated based on their ability to inhibit endogenous ethylene biosynthesis. Experimental kiwifruit were further examined to determine their ability to recover from ripening inhibition by a short treatment with exogenous ethylene. Kiwifruit cold-stored for 4 months and maintained for 8 d at 20 °C were treated with 100 μL L^− 1^ exogenous ethylene (20 °C, 90% RH, 24 h). Exogenous ethylene treatment was repeated after 6 months of cold storage plus 1 day of ripening in all treatments to validate the results obtained following 4 months of cold storage. Exogenous ethylene was applied in an airtight tank (100 L) with an internal fan to circulate air. Carbon dioxide was absorbed in a 500 mL solution of 4 M NaOH. Following the 24-h exposure, the ethylene concentration in the tank was 102 and 98 μL L^− 1^ and the CO_2_ concentration was 0.45 and 0.39% after 4 and 6 months of cold storage, respectively.

Kiwifruit ripening following cold storage was characterized using ethylene emission rate; CO_2_ emission rate (respiration rate, RR); pericarp and core tissue firmness; DMC, SSC and TA, as described [4]. Additionally, pericarp tissue was sampled from each biological replicate per treatment (3 replicates of pericarp flesh tissue consisted of 10 fruits each), frozen immediately in liquid nitrogen, and finally stored for further analysis (*−* 80 *°*C). In summary, kiwifruits in the present study were exposed to 4 postharvest treatments, namely, control, 1-MCP, O_3_ and 1-MCP + O_3_. In addition, a separate treatment with exogenous ethylene (ETH) exposure to postharvest treatments was applied after 4 and 6 months of cold storage, as presented schematically in Additional file 10: Figure S6.

### Kiwifruit ripening physico-chemical changes

Firmness of pericarp and core tissue was determined using a fruit texture analyzer (model 53,205, T.R. Turoni srl, Forlì, Italy) and expressed as newtons (N) following the methodology previously described [4]. Soluble solids concentration and TA were assessed as described [4]. Dry matter content was measured in 2 mm thin cylindrical slices from 3 biological replications of 10 fruits as described [44]. Mean values of physico-chemical data (3 biological repetitions) were subjected to analysis of variance (ANOVA) and least significant differences (LSD) (*P* = 0.05) calculated using the statistics package SPSS 22.0 for Mac OS X (SPSS, Chicago, IL, USA).

### Ethylene emission and respiration rates

Ethylene emission and respiration rates (RR) in 3 repetitions of 3 kiwifruit per treatment during ripening were analyzed using a gas chromatograph system and an infrared gas analyzer, respectively, as previously described [17]. Statistical analysis was as described above.

### Analysis of ethylene biosynthesis intermediates and enzyme activities

1-Aminocyclopropane-1-carboxylic acid (ACC) and 1-malonyl-aminocyclo-propane-1-carboxylic acid (MACC) concentrations and ACC synthase (ACS) and ACC oxidase (ACO) enzyme activities were analyzed as previously described [45]. Statistical analysis was as described above.

### Kiwifruit soluble protein extraction, 2D-gel electrophoresis and image quantification

Soluble proteins of kiwifruit pericarp tissue were extracted as previously described [46]. Protein concentrations were measured according to Bradford’s method [47]. First and second dimension separation of protein extracts (50 μg) was performed in three biological replications per treatment as described [4]. 2D-gels staining and scanning, and image quantification and analysis performed as previously described [4, 48]. Quantitative protein spot abundance mean values comparisons were performed by one-way analysis of variance using Student’s t-test (*P* = *0.05*). The statistically different means were further subjected to a quantitative criterion of 1.5-fold change for significant differences determination (Additional file 11: Table S5).

### Protein in-gel digestion and identification by mass spectrometry

Selected spots of interest on the 2D-gels were stained, isolated and tripsin digested as described [4, 19, 49]. Database searches were conducted against the Cornell University kiwifruit protein database (http://bioinfo.bti.cornell.edu/cgi-bin/kiwi/download.cgi) containing 39,004 protein sequences and the National Center for Biotechnology Information (NCBI) databases using BLASTp analyses and MASCOT software as described [4, 19]. Validation of significant differences through the two-way hierarchical clustering was done with Permut Matrix software. Zero-mean and unit-standard deviation was used for the row-by-row normalization of data. Analysis was performed using Pearson’s distance and Ward’s algorithm. Identification criteria, among the positive matches, included at least 2 different peptide sequences of over 6 amino acids with an individual score above 20 (identity peptide score corresponding to *P* < 0.05 was 18 for search in KIWIFRUIT GENOME); in some cases, manual protein BLAST was performed against the current databases as described [4, 19]. For the identifications based on a single peptide, the identity score was required, and additional information was provided as described previously [4, 19]. Details regarding the information of protein identification is provided (Additional file 4: Table S1). Identified kiwifruit proteins were categorized into functional classes as described [50].

### RNA isolation and reverse transcription quantitative real-time PCR (RT-qPCR) analysis

Total RNA was isolated and cDNA synthesis was performed as described [19, 51]. Target cDNAs amplification was performed with gene-specific primers (Additional file 12: Table S6) designed as described [19]. Quantitative RT-PCR reactions were performed as described [19]. The specificity of the primers was determined by the dissociation kinetics for the PCR products at the end of each run. Actin (*A. deliciosa*) was used as reference gene. Relative transcription of the gene of interest and PCR efficiency were calculated as described [19, 52]. Three biological replicates of each treatment were performed and used for gene expression experiments.

### Protein-protein interaction network bioinformatic analysis

A protein-protein interaction network (PPI) analysis and prediction of the potential biological processes and molecular functions was performed to shed light on interaction functions of identified proteins as described [4, 20, 21]. Search parameters and statistical analysis of the annotated protein entries for PPI and biological processes and molecular functions analysis were obtained by blasting identified proteins against TAIR10 (The *Arabidopsis* Information Resource). Annotated proteins with the highest score and lowest E-value were considered relevant for each identified protein (Additional file 9: Table S4) [4, 20, 21].

## Additional files

Additional file 1: **Figure S1.** Changes in dry matter content (DMC) in kiwifruit during ripening at 20 °C following 2 or 6 months of cold storage (0 °C, RH 90%). Vertical lines indicate LSD (*P* = 0.05) of three replicate samples, each consisting of 10 cylindrical slices coming from 10 separate fruit. (PPTX 85 kb)

Additional file 2: **Figure S2.** Soluble solids concentration (SSC, a, c, e) and titratable acidity (TA, b, d, f) in 1-MCP/O_3_-treated kiwifruit during ripening at 20 °C following 2, 4 or 6 months of cold storage (0 °C, RH 90%). Vertical lines indicate LSD (*P* = 0.05) of three replicate samples, each consisting of 10 fruit. (PPTX 110 kb)

Additional file 3: **Figure S3.** Soluble solids concentration (SSC, a, c) and titratable acidity (TA, b, d) in 1-MCP/O_3_/ETH-treated kiwifruit during ripening at 20 °C following 4 or 6 months of cold storage (0 °C, RH 90%). Vertical lines indicate LSD (*P* = 0.05) of three replicate samples, each consisting of 10 fruit. Markers and lines in grey represent kiwifruit untreated with exogenous ethylene as in Additional file 2: Figure S2. (PPTX 114 kb)

Additional file 4: **Table S1.** Information data and responsiveness to 1-MCP, O_3_ and exogenous ethylene treatments of identified kiwifruit proteins analyzed by 2DE-PAGE and nanoLC-MS/MS. (XLSX 29 kb)

Additional file 5: **Figure S4.** Reference maps for kiwifruit proteins per treatment. (PPTX 463 kb)

Additional file 6: **Figure S5.** Venn diagram showing the overlapping and unique kiwifruit proteins quantified in each single treatment exposed to exogenous ethylene (control-ETH, 1-MCP-ETH, O_3_-ETH, 1-MCP + O_3_-ETH) compared to their counterparts untreated with ethylene. (PPTX 149 kb)

Additional file 7: **Table S2. (a)** Biological pathways and networks analysis generated by BiNGO for proteins with altered abundance in kiwifruit treated with ripening inhibitors but not with exogenous ethylene. **(b)** Biological pathways and networks analysis generated by BiNGO for proteins with altered abundance in kiwifruit undergoing ripening with exogenous ethylene. (XLSX 15 kb)

Additional file 8: **Table S3. (a)** Molecular functions and networks analysis generated by BiNGO for proteins with altered abundance in kiwifruit treated with ripening inhibitors but not with exogenous ethylene. **(b)** Molecular functions and networks analysis generated by BiNGO for proteins with altered abundance in kiwifruit undergoing ripening with exogenous ethylene. (XLSX 12 kb)

Additional file 9: **Table S4.** Identified proteins of kiwifruit that Blasted against the TAIR database and their STRING 9.0 ID. (XLSX 21 kb)

Additional file 10: **Figure S6.** Experimental design. (PPTX 46 kb)

Additional file 11: **Table S5.** Quantitative data of protein spot volumes in kiwifruit on 2DE-PAGE gels. (XLSX 94 kb)

Additional file 12: **Table S6.** Primers used to perform q-RT PCR analysis. (DOCX 20 kb)

## Abbreviations

1-MCP: 1-methylcyclopropene
ACC: 1-aminocyclopropane-1-carboxylic acid
ACO: ACC oxidase
ACO1: 1-aminocyclopropane-1-carboxylic acid oxidase
ACS: ACC synthase
DMC: Dry matter content
ETR1: Ethylene receptor
EXP: Expansin
GGP1: Geranylgeranyl diphosphate synthase
LOX1: Lipoxygenase
MACC: 1-malonyl-aminocyclo-propane-1-carboxylic acid
MS/MS: Mass spectrometry
NCBI: National Center for Biotechnology Information
O_3_: Ozone
PG: Polygalacturonase
qRT-PCR: Quantitative reverse transcriptase polymerase chain reaction
SSC: Soluble solids concentration
TA: Titratable acidity

## References

[1] Gapper NE, McQuinn RP, Giovannoni JJ (2013). Molecular and genetic regulation of fruit ripening. Plant Mol Biol.

[2] Molassiotis A, Tanou G, Filippou P, Fotopoulos V (2013). Proteomics in the fruit tree science arena: new insights into fruit defence, development, and ripening. Proteomics.

[3] Atkinson RG, Gunaseelan K, Wang MY, Luo LK, Wang TC, Norling CL (2011). Dissecting the role of climacteric ethylene in kiwifruit (*Actinidia chinensis*) ripening using a 1-aminocyclopropane-1-carboxylic acid oxidase knockdown line. J Exp Bot.

[4] Minas IS, Tanou G, Karagiannis E, Belghazi M, Molassiotis A (2016). Coupling of physiological and proteomic analysis to understand the ethylene- and chilling-induced kiwifruit ripening syndrome. Front Plant Sci.

[5] Sisler EC, Serek M (1997). Inhibitors of ethylene responses in plants at the receptor level: recent developments. Physiol Plant.

[6] Watkins CB (2006). The use of 1-methylcyclopropene (1-MCP) on fruits and vegetables. Biotechnol Adv.

[7] Minas IS, Forcada CF, Dangl GS, Gradziel TM, Dandekar AM, Crisosto CH (2015). Discovery of non-climacteric and suppressed climacteric bud sport mutations originating from a climacteric Japanese plum cultivar (*Prunus salicina* lindl.). Front Plant Sci.

[8] Yin X-R, Allan AC, Chen K, Ferguson IB (2010). Kiwifruit EIL and ERF genes involved in regulating fruit ripening. Plant Physiol.

[9] Yin X, Chen K, Allan AC, Wu R, Zhang B, Lallu N (2008). Ethylene-induced modulation of genes associated with the ethylene signalling pathway in ripening kiwifruit. J Exp Bot.

[10] Mworia EG, Yoshikawa T, Salikon N, Oda C, Asiche WO, Yokotani N (2012). Low-temperature-modulated fruit ripening is independent of ethylene in “Sanuki gold” kiwifruit. J Exp Bot.

[11] Boquete EJ, Trinchero GD, Fraschina AA, Vilella F, Sozzi GO (2004). Ripening of ‘Hayward’ kiwifruit treated with 1-methylcyclopropene after cold storage. Postharvest Biol Technol.

[12] Ilina N, Alem HJ, Pagano EA, Sozzi GO (2010). Suppression of ethylene perception after exposure to cooling conditions delays the progress of softening in ‘Hayward’ kiwifruit. Postharvest Biol Technol.

[13] Rice RG (2002). Century 21 - pregnant with ozone. Ozone Sci Eng.

[14] Martínez-Romero D, Bailén G, Serrano M, Guillén F, Valverde JM, Zapata P (2007). Tools to maintain postharvest fruit and vegetable quality through the inhibition of ethylene action: a review. Crit Rev Food Sci Nutr.

[15] Tzortzakis N, Chrysargyris A (2017). Postharvest ozone application for the preservation of fruits and vegetables. Food Rev Int.

[16] Minas IS, Karaoglanidis GS, Manganaris GA, Vasilakakis M (2010). Effect of ozone application during cold storage of kiwifruit on the development of stem-end rot caused by *Botrytis cinerea*. Postharvest Biol Technol.

[17] Minas IS, Tanou G, Belghazi M, Job D, Manganaris GA, Molassiotis A (2012). Physiological and proteomic approaches to address the active role of ozone in kiwifruit post-harvest ripening. J Exp Bot.

[18] Minas IS, Vicente AR, Dhanapal AP, Manganaris GA, Goulas V, Vasilakakis M (2014). Ozone-induced kiwifruit ripening delay is mediated by ethylene biosynthesis inhibition and cell wall dismantling regulation. Plant Sci.

[19] Tanou G, Minas IS, Karagiannis E, Tsikou D, Audebert S, Papadopoulou KK (2015). The impact of sodium nitroprusside and ozone in kiwifruit ripening physiology: a combined gene and protein expression profiling approach. Ann Bot.

[20] Szklarczyk D, Franceschini A, Kuhn M, Simonovic M, Roth A, Minguez P (2011). The STRING database in 2011: functional interaction networks of proteins, globally integrated and scored. Nucleic Acids Res.

[21] Maere S, Heymans K, Kuiper M (2005). BiNGO: a Cytoscape plugin to assess overrepresentation of gene ontology categories in biological networks. Bioinformatics.

[22] Antunes MDC, Sfakiotakis EM (2002). Chilling induced ethylene biosynthesis in ‘Hayward’ kiwifruit following storage. Sci Hortic.

[23] Golding J, Shearer D, Wyllie S, McGlasson W (1998). Application of 1-MCP and propylene to identify ethylene-dependent ripening processes in mature banana fruit. Postharvest Biol Technol.

[24] Novikova GV, Moshkov IE, Smith AR, Hall MA (2003). Nucleoside diphosphate kinase is a possible component of the ethylene signal transduction pathway. Biochemistry.

[25] Huerta-Ocampo JÁ, Osuna-Castro JA, Lino-López GJ, Barrera-Pacheco A, Mendoza-Hernández G, De León-Rodríguez A (2012). Proteomic analysis of differentially accumulated proteins during ripening and in response to 1-MCP in papaya fruit. J Proteome.

[26] Kesari R, Trivedi PK, Nath P (2010). Gene expression of pathogenesis-related protein during banana ripening and after treatment with 1-MCP. Postharvest Biol Technol.

[27] Nardozza S, Boldingh HL, Osorio S, Höhne M, Wohlers M, Gleave AP (2013). Metabolic analysis of kiwifruit (*Actinidia deliciosa*) berries from extreme genotypes reveals hallmarks for fruit starch metabolism. J Exp Bot.

[28] Sabehat A, Lurie S, Weiss D (1998). Expression of small heat-shock proteins at low temperatures. A possible role in protecting against chilling injuries. Plant Physiol.

[29] Shiu S-H, Karlowski WM, Pan R, Tzeng Y-H, Mayer KFX, Li W-H (2004). Comparative analysis of the receptor-like kinase family in Arabidopsis and rice. Plant Cell.

[30] Radauer C, Lackner P, Breiteneder H (2008). The Bet v 1 fold: an ancient, versatile scaffold for binding of large, hydrophobic ligands. BMC Evol Biol.

[31] Wang X, Chang L, Tong Z, Wang D, Yin Q, Wang D (2016). Proteomics profiling reveals carbohydrate metabolic enzymes and 14-3-3 proteins play important roles for starch accumulation during cassava root tuberization. Sci Rep.

[32] Rocco M, D’Ambrosio C, Arena S, Faurobert M, Scaloni A, Marra M (2006). Proteomic analysis of tomato fruits from two ecotypes during ripening. Proteomics.

[33] Bianco L, Lopez L, Scalone AG, Di Carli M, Desiderio A, Benvenuto E (2009). Strawberry proteome characterization and its regulation during fruit ripening and in different genotypes. J Proteome.

[34] Pedreschi R, Lurie S, Hertog M, Nicolaï B, Mes J, Woltering E (2013). Post-harvest proteomics and food security. Proteomics.

[35] Etienne A, Génard M, Lobit P, Mbeguié-A-Mbéguié D, Bugaud C (2013). What controls fleshy fruit acidity? A review of malate and citrate accumulation in fruit cells. J Exp Bot.

[36] Xue Z, Liu X, Pang Y, Yu T, Xiao R, Jin M (2012). Characterization, phylogenetic analysis and cDNA cloning of natterin-like gene from the blood of lamprey. Immunol Lett.

[37] Miernyk JA, Thelen JJ (2008). Biochemical approaches for discovering protein-protein interactions. Plant J.

[38] Zegzouti H, Jones B, Frasse P, Marty C, Maitre B, Latch A (1999). Ethylene-regulated gene expression in tomato fruit: characterization of novel ethylene-responsive and ripening-related genes isolated by differential display. Plant J.

[39] Van Schie CCN, Ament K, Schmidt A, Lange T, Haring MA, Schuurink RC (2007). Geranyl diphosphate synthase is required for biosynthesis of gibberellins. Plant J.

[40] Cosgrove DJ (2015). Plant expansins: diversity and interactions with plant cell walls. Curr Opin Plant Biol.

[41] Rose JK, Bennett AB (1999). Cooperative disassembly of the cellulose–xyloglucan network of plant cell walls: parallels between cell expansion and fruit ripening. Trends Plant Sci.

[42] Pilati S, Brazzale D, Guella G, Milli A, Ruberti C, Biasioli F (2014). The onset of grapevine berry ripening is characterized by ROS accumulation and lipoxygenase-mediated membrane peroxidation in the skin. BMC Plant Biol.

[43] Karagiannis E, Tanou G, Samiotaki M, Michailidis M, Diamantidis G, Minas IS (2016). Comparative physiological and proteomic analysis reveal distinct regulation of peach skin quality traits by altitude. Front Plant Sci.

[44] Crisosto C, Hasey J, Cantin C, Garibay S, Crisosto G (2008). New kiwifruit dry weight protocol. UC Coop Ext Cent Val Postharvest Newsl.

[45] Bulens I, Van de Poel B, Hertog ML, De Proft MP, Geeraerd AH, Nicolaï BM (2011). Protocol: an updated integrated methodology for analysis of metabolites and enzyme activities of ethylene biosynthesis. Plant Methods.

[46] Tanou G, Filippou P, Belghazi M, Job D, Diamantidis G, Fotopoulos V (2012). Oxidative and nitrosative-based signaling and associated post-translational modifications orchestrate the acclimation of citrus plants to salinity stress. Plant J.

[47] Bradford MM (1976). A rapid and sensitive method for the quantitation of microgram quantities of protein utilizing the principle of protein-dye binding. Anal Biochem.

[48] Tanou G, Job C, Rajjou L, Arc E, Belghazi M, Diamantidis G (2009). Proteomics reveals the overlapping roles of hydrogen peroxide and nitric oxide in the acclimation of citrus plants to salinity. Plant J.

[49] Vu Hai V, Pages F, Boulanger N, Audebert S, Parola P, Almeras L (2013). Immunoproteomic identification of antigenic salivary biomarkers detected by *Ixodes ricinus*-exposed rabbit sera. Ticks Tick Borne Dis.

[50] Bevan M, Bancroft I, Bent E, Love K, Goodman H, Dean C (1998). Analysis of 1.9 Mb of contiguous sequence from chromosome 4 of *Arabidopsis thaliana*. Nature.

[51] Chang S, Puryear J, Cairney J (1993). A simple and efficient method for isolating RNA from pine trees. Plant Mol Biol Report.

[52] Ramakers C, Ruijter JM, Deprez RHL, Moorman AF (2003). Assumption-free analysis of quantitative real-time polymerase chain reaction (PCR) data. Neurosci Lett.

