# Supplementary figures and images for: Ozone-induced inhibition of kiwifruit ripening is amplified by 1-methylcyclopropene and reversed by exogenous ethylene
Source: BMC Plant Biol. 2018 Dec 17;18:358. doi: 10.1186/s12870-018-1584-y (PMC6296049; doi:10.1186/s12870-018-1584-y)

## Slide 1
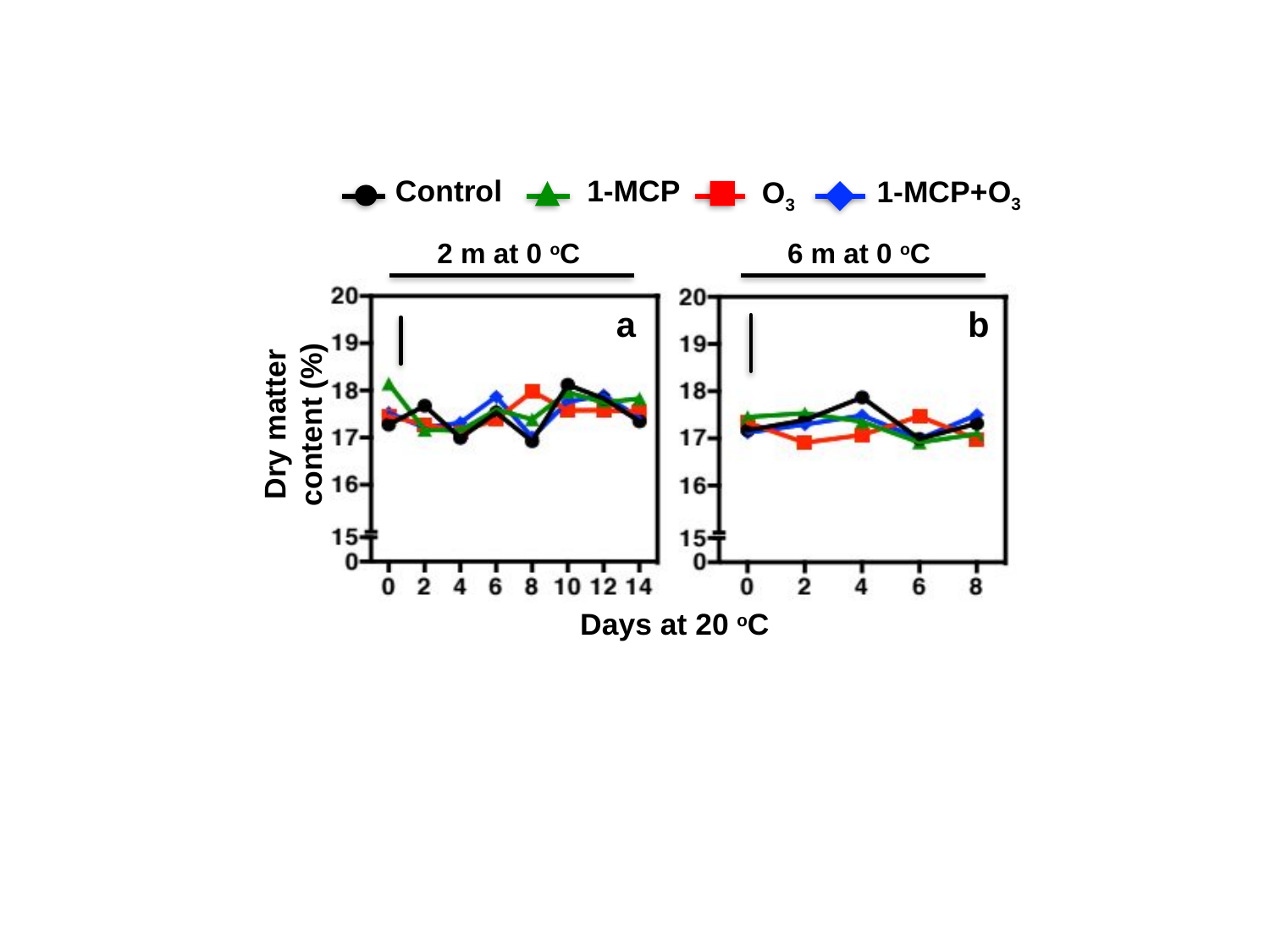

Control
1-MCP
1-MCP+O3
O3
2 m at 0 οC
6 m at 0 οC
a
b
Dry matter content (%)
Days at 20 οC

Supplement: Supplementary file 1 — Figure S1. Changes in dry matter content (DMC) in kiwifruit during ripening at 20 °C following 2 or 6 months of cold storage (0 °C, RH 90%). Vertical lines indicate LSD (P = 0.05) of three replicate samples, each consisting of 10 cylindrical slices coming from 10 separate fruit. (PPTX 85 kb) [file 12870_2018_1584_MOESM1_ESM.pptx]

## Slide 1
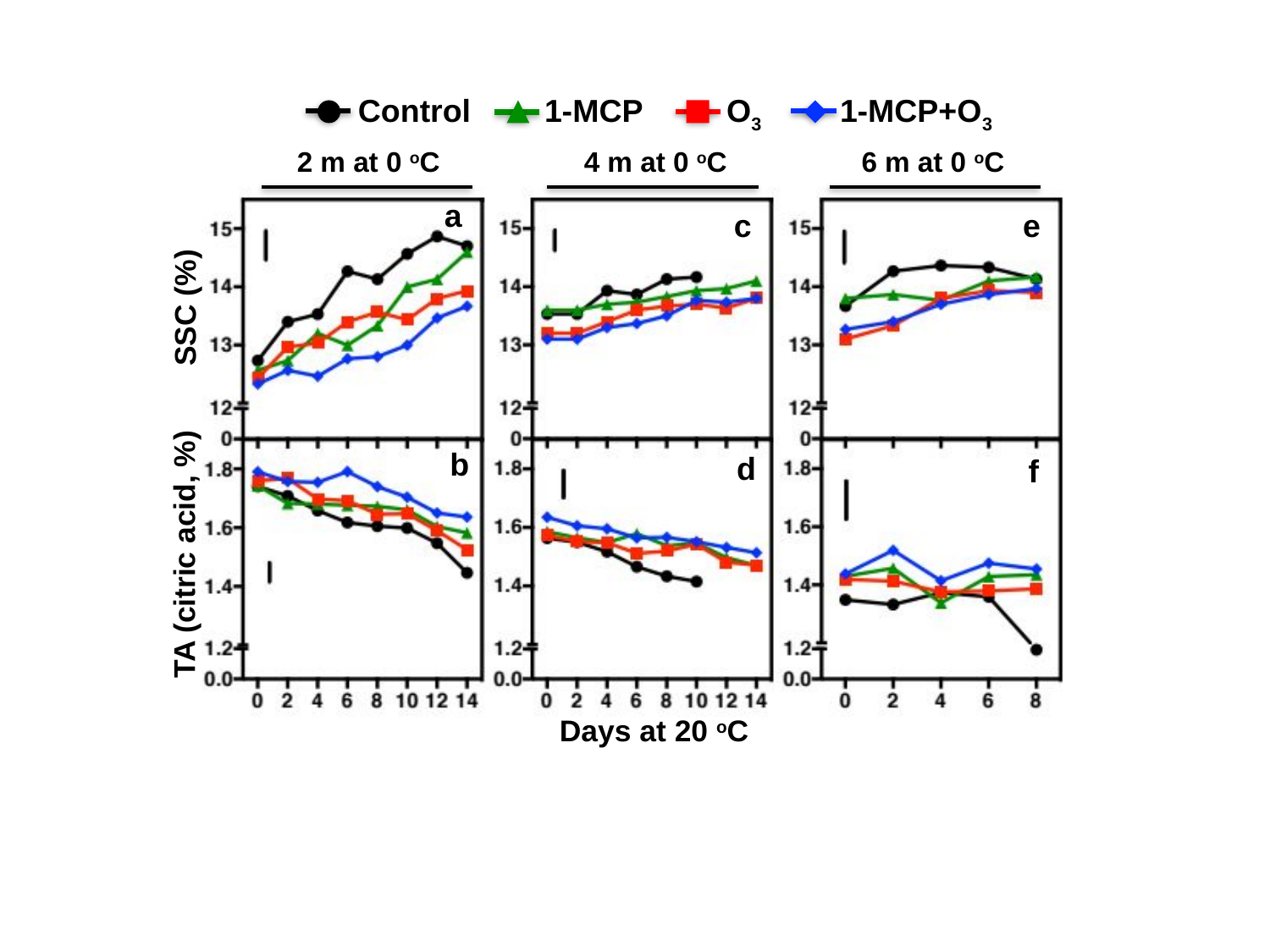

Control
1-MCP
O3
1-MCP+O3
2 m at 0 οC
4 m at 0 οC
6 m at 0 οC
a
c
e
SSC (%)
b
d
f
TA (citric acid, %)
Days at 20 οC

Supplement: Supplementary file 2 — Figure S2. Soluble solids concentration (SSC, a, c, e) and titratable acidity (TA, b, d, f) in 1-MCP/O3-treated kiwifruit during ripening at 20 °C following 2, 4 or 6 months of cold storage (0 °C, RH 90%). Vertical lines indicate LSD (P = 0.05) of three replicate samples, each consisting of 10 fruit. (PPTX 110 kb) [file 12870_2018_1584_MOESM2_ESM.pptx]

## Slide 1
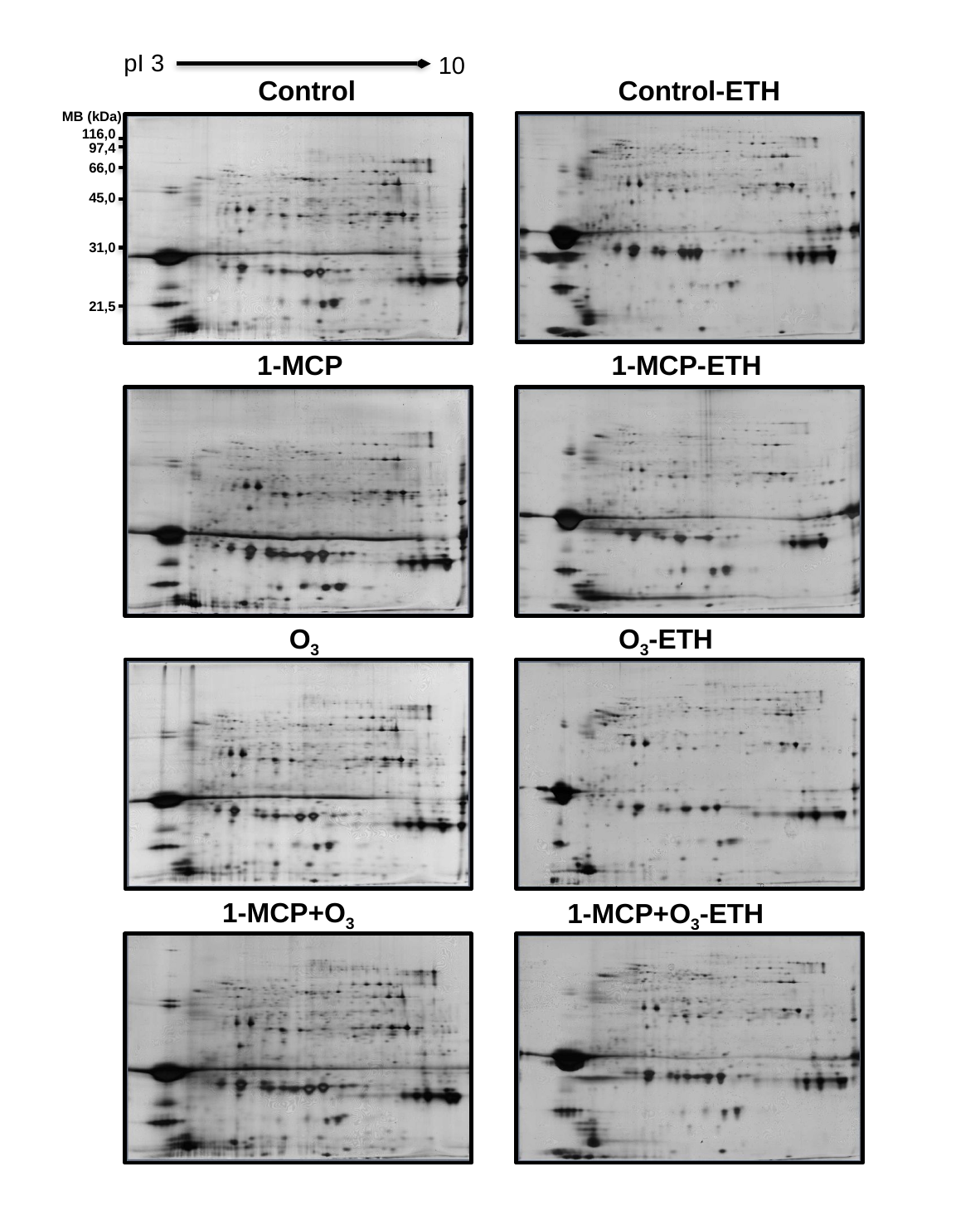

pΙ 3
10
Control
Control-ΕΤΗ
1-MCP
1-MCP-ΕΤΗ
O3
O3-ΕΤΗ
1-MCP+O3
1-MCP+O3-ΕΤΗ
ΜΒ (kDa)
116,0
97,4
66,0
45,0
31,0
21,5

Supplement: Supplementary file 5 — Figure S4. Reference maps for kiwifruit proteins per treatment. (PPTX 463 kb) [file 12870_2018_1584_MOESM5_ESM.pptx]
