# Supplementary material for: Ozone-induced inhibition of kiwifruit ripening is amplified by 1-methylcyclopropene and reversed by exogenous ethylene
Source: BMC Plant Biol. 2018 Dec 17;18:358. doi: 10.1186/s12870-018-1584-y (PMC6296049; doi:10.1186/s12870-018-1584-y)
Supplement: Supplementary file 3 — Figure S3. Soluble solids concentration (SSC, a, c) and titratable acidity (TA, b, d) in 1-MCP/O3/ETH-treated kiwifruit during ripening at 20 °C following 4 or 6 months of cold storage (0 °C, RH 90%). Vertical lines indicate LSD (P = 0.05) of three replicate samples, each consisting of 10 fruit. Markers and lines in grey represent kiwifruit untreated with exogenous ethylene as in Additional file 2: Figure S2. (PPTX 114 kb) [file 12870_2018_1584_MOESM3_ESM.pptx]

## Slide 1
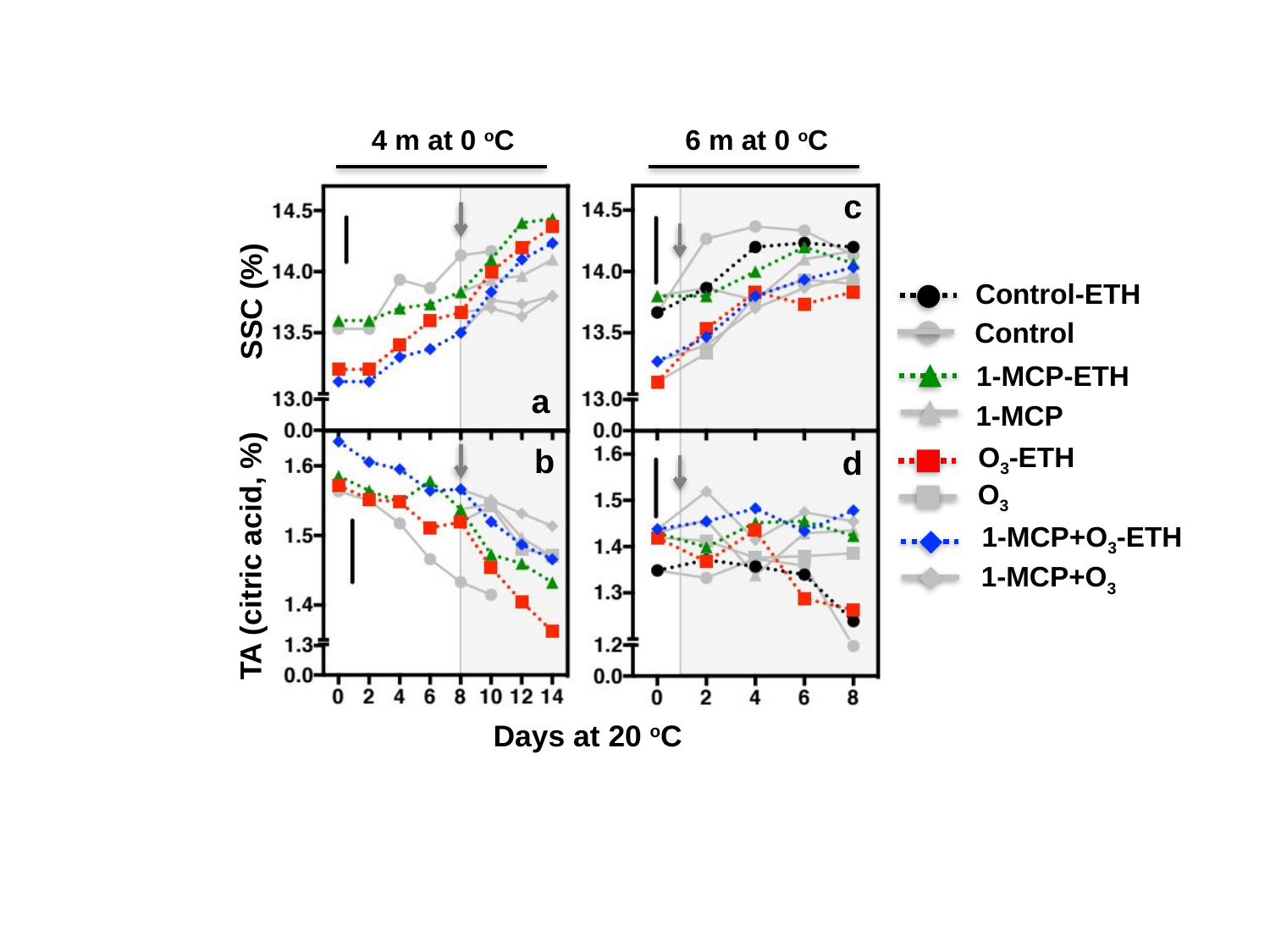

4 m at 0 οC
6 m at 0 οC
Days at 20 οC
c
Control-ETH
Control
1-MCP-ETH
1-MCP
O3-ETH
O3
1-MCP+O3-ETH
1-MCP+O3
SSC (%)
a
b
d
TA (citric acid, %)
