# Supplementary material for: Ozone-induced inhibition of kiwifruit ripening is amplified by 1-methylcyclopropene and reversed by exogenous ethylene
Source: BMC Plant Biol. 2018 Dec 17;18:358. doi: 10.1186/s12870-018-1584-y (PMC6296049; doi:10.1186/s12870-018-1584-y)
Supplement: Supplementary file 6 — Figure S5. Venn diagram showing the overlapping and unique kiwifruit proteins quantified in each single treatment exposed to exogenous ethylene (control-ETH, 1-MCP-ETH, O3-ETH, 1-MCP + O3-ETH) compared to their counterparts untreated with ethylene. (PPTX 149 kb) [file 12870_2018_1584_MOESM6_ESM.pptx]

## Slide 1
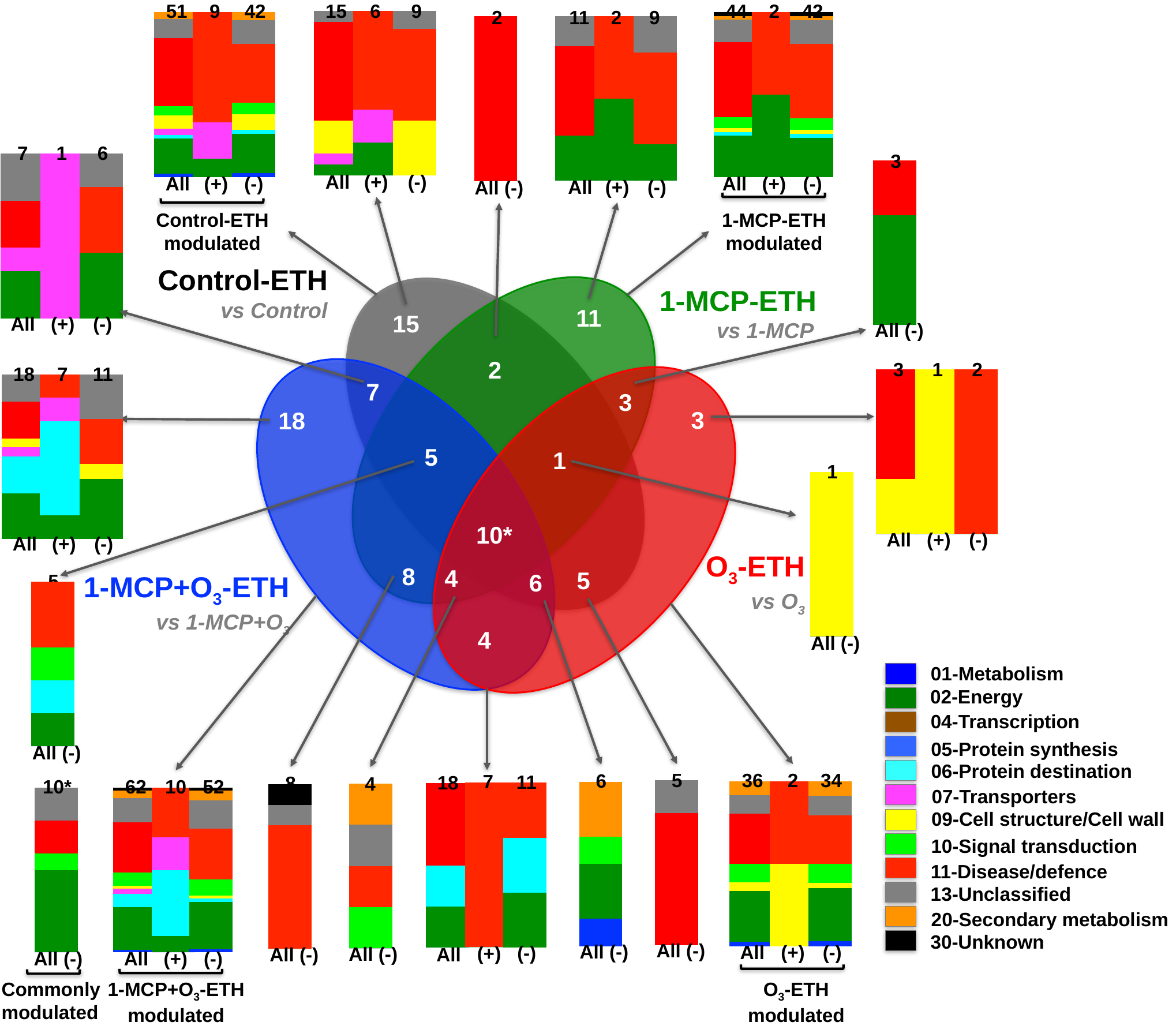

51
### Chart
| Category | 01-metabolism | 02-energy | 04-transription | 05-protein synthesis | 06-protein destination | 07-transporters | 09-cell structure | 10-signal transduction | 11-disease_defence | 13-unclassified | 20-secondary metabolism | 30-unknown |
|---|---|---|---|---|---|---|---|---|---|---|---|---|All
9
### Chart
| Category | 01-metabolism | 02-energy | 04-transription | 05-protein synthesis | 06-protein destination | 07-transporters | 09-cell structure | 10-signal transduction | 11-disease_defence | 13-unclassified | 20-secondary metabolism | 30-unknown |
|---|---|---|---|---|---|---|---|---|---|---|---|---|(+)
42
### Chart
| Category | 01-metabolism | 02-energy | 04-transription | 05-protein synthesis | 06-protein destination | 07-transporters | 09-cell structure | 10-signal transduction | 11-disease_defence | 13-unclassified | 20-secondary metabolism | 30-unknown |
|---|---|---|---|---|---|---|---|---|---|---|---|---|(-)
15
### Chart
| Category | 01-metabolism | 02-energy | 04-transription | 05-protein synthesis | 06-protein destination | 07-transporters | 09-cell structure | 10-signal transduction | 11-disease_defence | 13-unclassified | 20-secondary metabolism | 30-unknown |
|---|---|---|---|---|---|---|---|---|---|---|---|---|All
6
### Chart
| Category | 01-metabolism | 02-energy | 04-transription | 05-protein synthesis | 06-protein destination | 07-transporters | 09-cell structure | 10-signal transduction | 11-disease_defence | 13-unclassified | 20-secondary metabolism | 30-unknown |
|---|---|---|---|---|---|---|---|---|---|---|---|---|(+)
9
### Chart
| Category | 01-metabolism | 02-energy | 04-transription | 05-protein synthesis | 06-protein destination | 07-transporters | 09-cell structure | 10-signal transduction | 11-disease_defence | 13-unclassified | 20-secondary metabolism | 30-unknown |
|---|---|---|---|---|---|---|---|---|---|---|---|---|(-)
44
### Chart
| Category | 01-metabolism | 02-energy | 04-transription | 05-protein synthesis | 06-protein destination | 07-transporters | 09-cell structure | 10-signal transduction | 11-disease_defence | 13-unclassified | 20-secondary metabolism | 30-unknown |
|---|---|---|---|---|---|---|---|---|---|---|---|---|All
2
### Chart
| Category | 01-metabolism | 02-energy | 04-transription | 05-protein synthesis | 06-protein destination | 07-transporters | 09-cell structure | 10-signal transduction | 11-disease_defence | 13-unclassified | 20-secondary metabolism | 30-unknown |
|---|---|---|---|---|---|---|---|---|---|---|---|---|(+)
42
### Chart
| Category | 01-metabolism | 02-energy | 04-transription | 05-protein synthesis | 06-protein destination | 07-transporters | 09-cell structure | 10-signal transduction | 11-disease_defence | 13-unclassified | 20-secondary metabolism | 30-unknown |
|---|---|---|---|---|---|---|---|---|---|---|---|---|(-)
2
### Chart
| Category | 01-metabolism | 02-energy | 04-transription | 05-protein synthesis | 06-protein destination | 07-transporters | 09-cell structure | 10-signal transduction | 11-disease_defence | 13-unclassified | 20-secondary metabolism | 30-unknown |
|---|---|---|---|---|---|---|---|---|---|---|---|---|All (-)
11
### Chart
| Category | 01-metabolism | 02-energy | 04-transription | 05-protein synthesis | 06-protein destination | 07-transporters | 09-cell structure | 10-signal transduction | 11-disease_defence | 13-unclassified | 20-secondary metabolism | 30-unknown |
|---|---|---|---|---|---|---|---|---|---|---|---|---|All
2
### Chart
| Category | 01-metabolism | 02-energy | 04-transription | 05-protein synthesis | 06-protein destination | 07-transporters | 09-cell structure | 10-signal transduction | 11-disease_defence | 13-unclassified | 20-secondary metabolism | 30-unknown |
|---|---|---|---|---|---|---|---|---|---|---|---|---|(+)
9
### Chart
| Category | 01-metabolism | 02-energy | 04-transription | 05-protein synthesis | 06-protein destination | 07-transporters | 09-cell structure | 10-signal transduction | 11-disease_defence | 13-unclassified | 20-secondary metabolism | 30-unknown |
|---|---|---|---|---|---|---|---|---|---|---|---|---|(-)
7
### Chart
| Category | 01-metabolism | 02-energy | 04-transription | 05-protein synthesis | 06-protein destination | 07-transporters | 09-cell structure | 10-signal transduction | 11-disease_defence | 13-unclassified | 20-secondary metabolism | 30-unknown |
|---|---|---|---|---|---|---|---|---|---|---|---|---|All
1
### Chart
| Category | 01-metabolism | 02-energy | 04-transription | 05-protein synthesis | 06-protein destination | 07-transporters | 09-cell structure | 10-signal transduction | 11-disease_defence | 13-unclassified | 20-secondary metabolism | 30-unknown |
|---|---|---|---|---|---|---|---|---|---|---|---|---|(+)
6
### Chart
| Category | 01-metabolism | 02-energy | 04-transription | 05-protein synthesis | 06-protein destination | 07-transporters | 09-cell structure | 10-signal transduction | 11-disease_defence | 13-unclassified | 20-secondary metabolism | 30-unknown |
|---|---|---|---|---|---|---|---|---|---|---|---|---|(-)
3
### Chart
| Category | 01-metabolism | 02-energy | 04-transription | 05-protein synthesis | 06-protein destination | 07-transporters | 09-cell structure | 10-signal transduction | 11-disease_defence | 13-unclassified | 20-secondary metabolism | 30-unknown |
|---|---|---|---|---|---|---|---|---|---|---|---|---|All (-)
Control-ETH modulated
1-MCP-ETH modulated
11
15
2
7
3
3
18
5
1
10*
8
4
5
6
4
Control-ΕΤH
vs Control
1-ΜCP-ΕΤH
 vs 1-MCP
3
### Chart
| Category | 01-metabolism | 02-energy | 04-transription | 05-protein synthesis | 06-protein destination | 07-transporters | 09-cell structure | 10-signal transduction | 11-disease_defence | 13-unclassified | 20-secondary metabolism | 30-unknown |
|---|---|---|---|---|---|---|---|---|---|---|---|---|All
1
### Chart
| Category | 01-metabolism | 02-energy | 04-transription | 05-protein synthesis | 06-protein destination | 07-transporters | 09-cell structure | 10-signal transduction | 11-disease_defence | 13-unclassified | 20-secondary metabolism | 30-unknown |
|---|---|---|---|---|---|---|---|---|---|---|---|---|(+)
2
### Chart
| Category | 01-metabolism | 02-energy | 04-transription | 05-protein synthesis | 06-protein destination | 07-transporters | 09-cell structure | 10-signal transduction | 11-disease_defence | 13-unclassified | 20-secondary metabolism | 30-unknown |
|---|---|---|---|---|---|---|---|---|---|---|---|---|(-)
18
### Chart
| Category | 01-metabolism | 02-energy | 04-transription | 05-protein synthesis | 06-protein destination | 07-transporters | 09-cell structure | 10-signal transduction | 11-disease_defence | 13-unclassified | 20-secondary metabolism | 30-unknown |
|---|---|---|---|---|---|---|---|---|---|---|---|---|All
7
### Chart
| Category | 01-metabolism | 02-energy | 04-transription | 05-protein synthesis | 06-protein destination | 07-transporters | 09-cell structure | 10-signal transduction | 11-disease_defence | 13-unclassified | 20-secondary metabolism | 30-unknown |
|---|---|---|---|---|---|---|---|---|---|---|---|---|(+)
11
### Chart
| Category | 01-metabolism | 02-energy | 04-transription | 05-protein synthesis | 06-protein destination | 07-transporters | 09-cell structure | 10-signal transduction | 11-disease_defence | 13-unclassified | 20-secondary metabolism | 30-unknown |
|---|---|---|---|---|---|---|---|---|---|---|---|---|(-)
1
### Chart
| Category | 01-metabolism | 02-energy | 04-transription | 05-protein synthesis | 06-protein destination | 07-transporters | 09-cell structure | 10-signal transduction | 11-disease_defence | 13-unclassified | 20-secondary metabolism | 30-unknown |
|---|---|---|---|---|---|---|---|---|---|---|---|---|All (-)
O3-ΕΤΗ
vs O3
1-ΜCP+O3-ΕΤΗ
 vs 1-MCP+O3
5
### Chart
| Category | 01-metabolism | 02-energy | 04-transription | 05-protein synthesis | 06-protein destination | 07-transporters | 09-cell structure | 10-signal transduction | 11-disease_defence | 13-unclassified | 20-secondary metabolism | 30-unknown |
|---|---|---|---|---|---|---|---|---|---|---|---|---|All (-)
01-Metabolism
02-Energy
04-Transcription
05-Protein synthesis
06-Protein destination
07-Transporters
09-Cell structure/Cell wall
10-Signal transduction
11-Disease/defence
13-Unclassified
20-Secondary metabolism
30-Unknown
5
### Chart
| Category | 01-metabolism | 02-energy | 04-transription | 05-protein synthesis | 06-protein destination | 07-transporters | 09-cell structure | 10-signal transduction | 11-disease_defence | 13-unclassified | 20-secondary metabolism | 30-unknown |
|---|---|---|---|---|---|---|---|---|---|---|---|---|All (-)
36
### Chart
| Category | 01-metabolism | 02-energy | 04-transription | 05-protein synthesis | 06-protein destination | 07-transporters | 09-cell structure | 10-signal transduction | 11-disease_defence | 13-unclassified | 20-secondary metabolism | 30-unknown |
|---|---|---|---|---|---|---|---|---|---|---|---|---|All
2
### Chart
| Category | 01-metabolism | 02-energy | 04-transription | 05-protein synthesis | 06-protein destination | 07-transporters | 09-cell structure | 10-signal transduction | 11-disease_defence | 13-unclassified | 20-secondary metabolism | 30-unknown |
|---|---|---|---|---|---|---|---|---|---|---|---|---|(+)
34
### Chart
| Category | 01-metabolism | 02-energy | 04-transription | 05-protein synthesis | 06-protein destination | 07-transporters | 09-cell structure | 10-signal transduction | 11-disease_defence | 13-unclassified | 20-secondary metabolism | 30-unknown |
|---|---|---|---|---|---|---|---|---|---|---|---|---|(-)
6
### Chart
| Category | 01-metabolism | 02-energy | 04-transription | 05-protein synthesis | 06-protein destination | 07-transporters | 09-cell structure | 10-signal transduction | 11-disease_defence | 13-unclassified | 20-secondary metabolism | 30-unknown |
|---|---|---|---|---|---|---|---|---|---|---|---|---|All (-)
7
### Chart
| Category | 01-metabolism | 02-energy | 04-transription | 05-protein synthesis | 06-protein destination | 07-transporters | 09-cell structure | 10-signal transduction | 11-disease_defence | 13-unclassified | 20-secondary metabolism | 30-unknown |
|---|---|---|---|---|---|---|---|---|---|---|---|---|(+)
11
### Chart
| Category | 01-metabolism | 02-energy | 04-transription | 05-protein synthesis | 06-protein destination | 07-transporters | 09-cell structure | 10-signal transduction | 11-disease_defence | 13-unclassified | 20-secondary metabolism | 30-unknown |
|---|---|---|---|---|---|---|---|---|---|---|---|---|(-)
18
### Chart
| Category | 01-metabolism | 02-energy | 04-transription | 05-protein synthesis | 06-protein destination | 07-transporters | 09-cell structure | 10-signal transduction | 11-disease_defence | 13-unclassified | 20-secondary metabolism | 30-unknown |
|---|---|---|---|---|---|---|---|---|---|---|---|---|All
8
### Chart
| Category | 01-metabolism | 02-energy | 04-transription | 05-protein synthesis | 06-protein destination | 07-transporters | 09-cell structure | 10-signal transduction | 11-disease_defence | 13-unclassified | 20-secondary metabolism | 30-unknown |
|---|---|---|---|---|---|---|---|---|---|---|---|---|All (-)
4
### Chart
| Category | 01-metabolism | 02-energy | 04-transription | 05-protein synthesis | 06-protein destination | 07-transporters | 09-cell structure | 10-signal transduction | 11-disease_defence | 13-unclassified | 20-secondary metabolism | 30-unknown |
|---|---|---|---|---|---|---|---|---|---|---|---|---|All (-)
10*
### Chart
| Category | 01-metabolism | 02-energy | 04-transription | 05-protein synthesis | 06-protein destination | 07-transporters | 09-cell structure | 10-signal transduction | 11-disease_defence | 13-unclassified | 20-secondary metabolism | 30-unknown |
|---|---|---|---|---|---|---|---|---|---|---|---|---|All (-)
62
### Chart
| Category | 01-metabolism | 02-energy | 04-transription | 05-protein synthesis | 06-protein destination | 07-transporters | 09-cell structure | 10-signal transduction | 11-disease_defence | 13-unclassified | 20-secondary metabolism | 30-unknown |
|---|---|---|---|---|---|---|---|---|---|---|---|---|All
10
### Chart
| Category | 01-metabolism | 02-energy | 04-transription | 05-protein synthesis | 06-protein destination | 07-transporters | 09-cell structure | 10-signal transduction | 11-disease_defence | 13-unclassified | 20-secondary metabolism | 30-unknown |
|---|---|---|---|---|---|---|---|---|---|---|---|---|(+)
52
### Chart
| Category | 01-metabolism | 02-energy | 04-transription | 05-protein synthesis | 06-protein destination | 07-transporters | 09-cell structure | 10-signal transduction | 11-disease_defence | 13-unclassified | 20-secondary metabolism | 30-unknown |
|---|---|---|---|---|---|---|---|---|---|---|---|---|(-)
Commonly modulated
1-MCP+O3-ETH modulated
O3-ETH modulated
