# Supplementary material for: Ozone-induced inhibition of kiwifruit ripening is amplified by 1-methylcyclopropene and reversed by exogenous ethylene
Source: BMC Plant Biol. 2018 Dec 17;18:358. doi: 10.1186/s12870-018-1584-y (PMC6296049; doi:10.1186/s12870-018-1584-y)
Supplement: Supplementary file 10 — Figure S6. Experimental design. (PPTX 46 kb) [file 12870_2018_1584_MOESM10_ESM.pptx]

## Slide 1
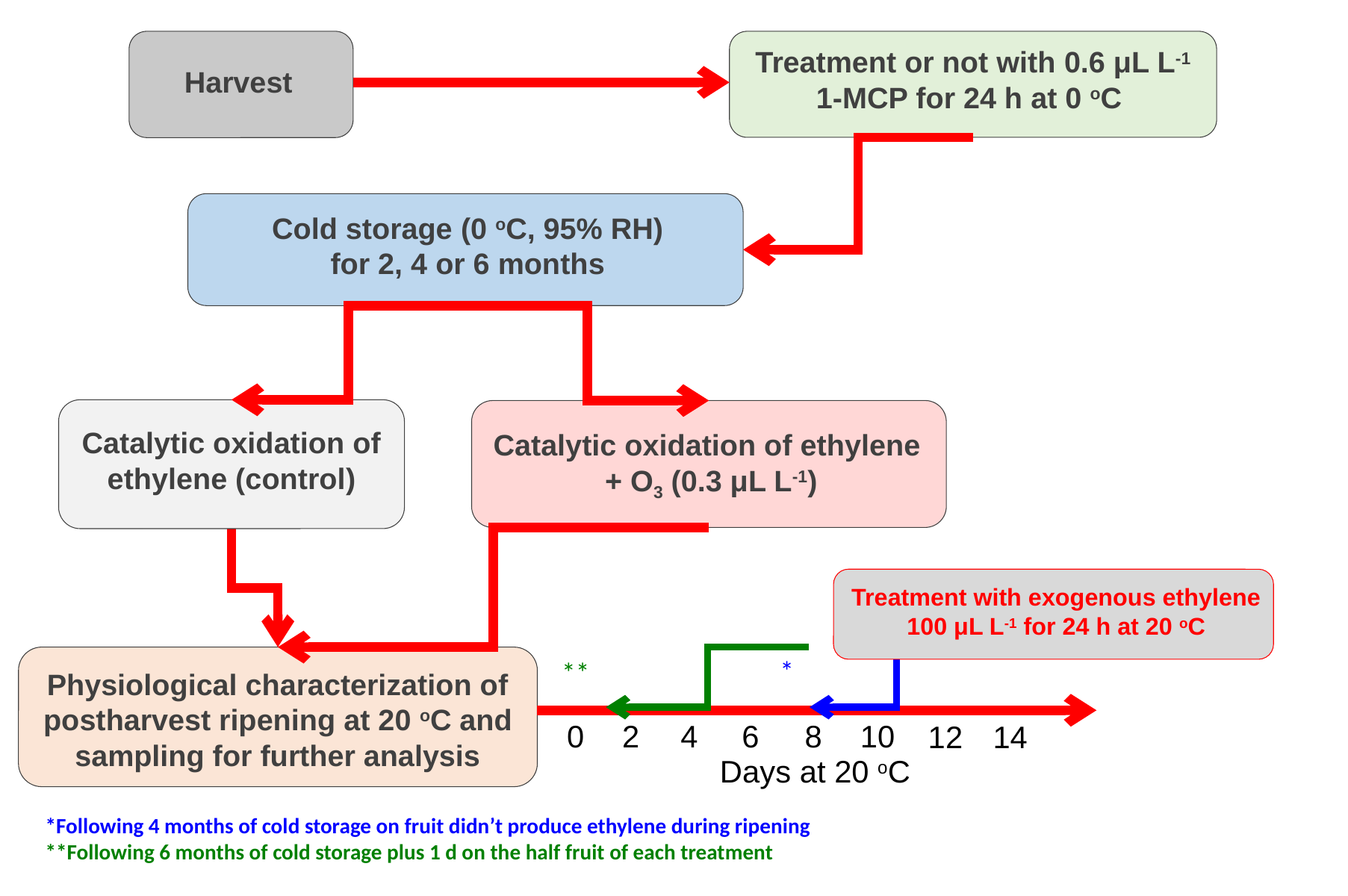

Harvest
Treatment or not with 0.6 μL L-1 1-MCP for 24 h at 0 οC
 Cold storage (0 οC, 95% RH)
 for 2, 4 or 6 months
Catalytic oxidation of ethylene (control)
Catalytic oxidation of ethylene
 + O3 (0.3 μL L-1)
Physiological characterization of postharvest ripening at 20 oC and sampling for further analysis
4
6
8
10
0
2
12
14
Days at 20 οC
Treatment with exogenous ethylene 100 μL L-1 for 24 h at 20 οC
*
**
*Following 4 months of cold storage on fruit didn’t produce ethylene during ripening
**Following 6 months of cold storage plus 1 d on the half fruit of each treatment
