# Supplementary material for: Ozone-induced inhibition of kiwifruit ripening is amplified by 1-methylcyclopropene and reversed by exogenous ethylene
Source: BMC Plant Biol. 2018 Dec 17;18:358. doi: 10.1186/s12870-018-1584-y (PMC6296049; doi:10.1186/s12870-018-1584-y)
Supplement: Supplementary file 12 — Table S6. Primers used to perform q-RT PCR analysis. (DOCX 20 kb) [file 12870_2018_1584_MOESM12_ESM.docx]

| **Target Gene** | **Forward Primer (5’-3’)** | **Reverse Primer (5’-3’)** |
| --- | --- | --- |
| Actin | CGAACGGGAAATTGTCCGT | TTCTCAACTGAGGAGCTGCTCTT |
| ACC oxidase (ACO1) | TGAGGTAATCACAAATGGCAAG | TGCTGATCTTCTTCTTTGTCCA |
| Ethylene receptor (ETR) | AACATGGCTAAGCTGGAATTTTGGC | AAAGCTGCTTCAGAAACTCGGGTG |
| Lipoxygenase (LOX1) | TCGGGTCAGAGATCAAAAGCA | GAAGCCTACGAGACAATCCATCAT |
| Geranylgeranyl diphosphate synthase (GGPS) | TCAAGACAGCAAAGCCATATATTTCAT | GCGTGTGGGGCCATATTAGGA |
| Expansin 2 (EXP2) | TCATTCCAAGGCCCCATT | AAGCACCTAAAACCAAAA |
| Polygalacturonase (PG) | TAGCGACGTGACGTATCAGG | TTCCTCCATCGTTTTCCTTG |

**Additional file 12:** **Table S6**. Primers used to perform qRT-PCR analysis.
